# Two Additional New Compounds from the Marine-Derived Fungus *Pseudallescheria ellipsoidea* F42-3

**DOI:** 10.3390/molecules21040442

**Published:** 2016-04-01

**Authors:** Kun-Teng Wang, Meng-Yang Xu, Wei Liu, Hou-Jin Li, Jun Xu, De-Po Yang, Wen-Jian Lan, Lai-You Wang

**Affiliations:** 1 School of Pharmacy, Guangdong Metabolic Diseases Research Center of Integrated Chinese and Western Medicine, Guangdong Pharmaceutical University, Guangzhou 510006, China; wangkunteng@sina.cn (K.-T.W.); wwl1430@outlook.com (W.L.); 2 School of Pharmaceutical Sciences, Sun Yat-sen University, Guangzhou 510006, China; xumy3@mail2.sysu.edu.cn (M.-Y.X.); junxu@biochemomes.com (J.X.); lssydp@mail.sysu.edu.cn (D.-P.Y.); lanwj@mail.sysu.edu.cn (W.-J.L.); 3 School of Chemistry and Chemical Engineering, Sun Yat-sen University, Guangzhou 510275, China; ceslhj@mail.sysu.edu.cn; 4 Guangdong Technology Research Center for Advanced Chinese Medicine, Guangzhou 510006, China

**Keywords:** *Pseudallescheria ellipsoidea*, diketopiperazine, lasiodiplodin, pseudellone, *Lobophytum crassum*

## Abstract

Two additional new compounds, pseudellone D (**1**) and (5*S*,6*S*)-dihydroxylasiodiplodin (**3**), along with the two known compounds lasiodipline F (**2**), (5*S*)-hydroxylasiodiplodin (**4**) were isolated from the marine-derived fungus *Pseudallescheria ellipsoidea* F42-3 associated with the soft coral *Lobophytum crassum*. Their structures, including absolute configurations, were elucidated on the basis of the corresponding spectroscopic data and electronic circular dichroism (ECD) spectra.

## 1. Introduction

In recent years, secondary metabolites from marine-derived fungi have drawn considerable attention for their unique structures and interesting biological properties [1]. In our continued exploration of the chemical potential of fungi from special niches, such as soft corals and sponges, a fungal strain characterized as *Pseudallescheria ellipsoidea* was isolated from the soft coral *Lobophytum crassum*. This fungus can cause invasive infections with a high mortality rate and is difficult to treat [2], but its chemistry is little explored. 

**Figure 1:**
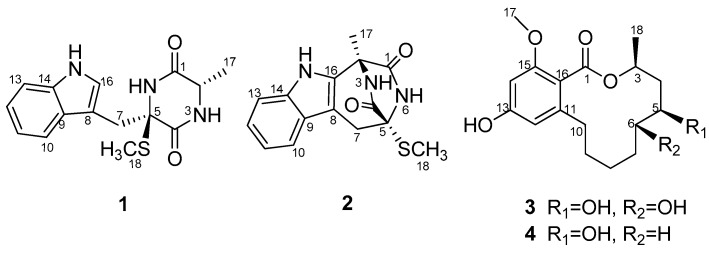
Chemical structures of compounds **1**–**4**.

Previously, we obtained three novel alkaloids, named pseudellones A–C, from the culture broth in glucose-peptone-yeast extract (GluPY) medium of the fungus *Pseudallescheria ellipsoidea* F42-3 [3]. The ongoing investigation on the secondary metabolites of this fungus afforded two additional new compounds: a diketopiperazine pseudellone D (**1**) and a 12-membered lactone derivative (5*S*,6*S*)-dihydroxylasiodiplodin (**3**), as well as two known related analogues: lasiodipline F (**2**), and (5*S*)-hydroxylasiodiplodin (**4**) (Figure 1) by silica gel column chromatography and RP-HPLC of the EtOAc extract of the culture broth. The structure elucidation was accomplished by MS, 1D, 2D NMR and ECD calculation. Herein, we describe the isolation and structure determination of the new compounds **1** and **2**.

## 2. Results and Discussion

**Figure 2:**
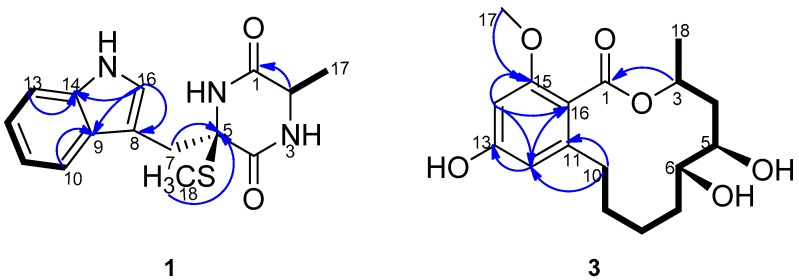
^1^H-^1^H COSY (bold line) and main HMBC (arrow) correlations of **1** and **3**.

**Figure 3:**
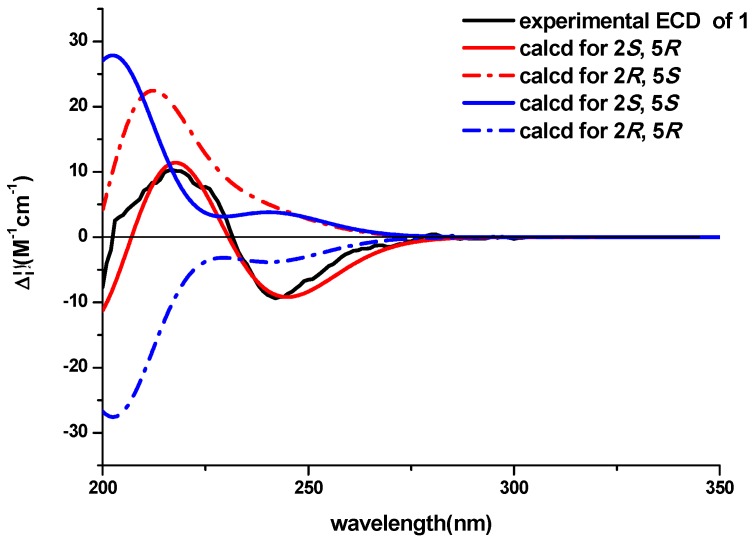
Comparison of the experimental ECD spectra of **1** with the calculated ECD spectra for four stereochemical options.

Pseudellone D (**1**) was obtained as a pale yellow solid. The molecular formula was established as C_15_H_17_N_3_O_2_S from a positive HREIMS peak at *m*/*z* 303.1029 [M]^+^ (calcd. 303.1041). The ^1^H- and ^13^C-NMR spectra (Table 1) displayed characteristic signals for a diketopiperazine. Two carbonyl groups at δ_C_ 166.5 and 169.3 along with the two α-amino acid carbon resonances at δ_C_ 51.9 and 68.1 and one α-amino acid methine proton at 3.44 (qd, 7.2, 2.4) implied the existence of two amino acid residues. The presence of an indole nucleus in the structure was deduced from the chemical shifts and the splitting pattern of the five aromatic protons at δ_H_ 7.76 (d, H-10), 6.99 (td, H-11), 7.07 (td, H-12), 7.35 (d, H-13), 7.24 (d, H-16) in the ^1^H-NMR spectrum, which was further confirmed from the ^1^H-^1^H COSY data and the HMBC crosspeaks from H-10 to C-9, from H-13 to C-14, from H-16 to C-8, C-9 and C-14 (Figure 2). Additionally, the ^1^H-NMR spectrum exhibited three exchangeable protons at δ_H_ 7.17, 7.50 and 10.18 attributed to two amide protons and an indole amino, and two methylene protons at δ_H_ 3.78 (d, H-7a), 3.21 (d, H-7b) together with S- and C-bonded methyl groups at δ_H_ 2.29(s, H_3_-18) and 1.35 (d, H_3_-17). Accordingly, the ^13^C-NMR and DEPT spectra showed fifteen carbon signals consisting of two carbonyl groups, eight aromatic carbons, one sp^3^ quaternary carbon, one sp^3^ methine, one sp^3^ methylene and two methyls. The ^1^H-^1^HCOSY correlation between H-2 and H_3_-17 and the HMBC crosspeaks from H-2 to C-1, H-7 to C-5 and C-8, and from H_3_-18 to C-5 determined the planar structure of the compound. The NOESY experiment didn’t provide enough information to assign the relative configuration of the compound. The ECD spectra with the four configuration options (2*S*,5*R*)-, (2*R*,5*S*)-, (2*R*,5*R*)- and -(2*S*,5*S*) resulting from the two chiral centers in the structure were calculated and compared with the experimental CD curve. As shown in Figure 3, the spectrum calculated for the (2*S*,5*R*)-stereochemistry agreed with the experimental one. Therefore, the absolute configuration of the compound was established to be (2*S*,5*R*). The known compound lasiodipline F (**2**) [4], was identified as the cyclization product of C-2 and C-16 of pseudellone D. The experimental CD spectrum of **2** reproduced (Figure S39) the experimental and computed CD curves of lasiodipline F in the literature. It was notable that the absolute stereochemistry of the two chiral centers of lasiodipline F was determined as (2*R*,5*S*), opposite of the (2*S*,5*R*) one of pseudellone D because of the configurational transformation occurring during the cyclization process.

(5*S*,6*S*)-Dihydroxylasiodiplodin (**3**) was isolated as a white solid. The molecular formula was established as C_17_H_24_O_6_ by the [M]^+^ peak 324.1562 in high-resolution EI mass spectrometry, indicating six degrees of unsaturation. Careful inspection of its ^1^H-, ^13^C- (Table 1), DEPT and HMQC spectra disclosed the existence of seventeen carbons, including one carbonyl, four sp^2^ quaternary carbons, two sp^2^ methines, three oxygenated sp^3^ methines, five sp^3^ methenes, one oxygenated sp^3^ methyl and one sp^3^ methyl. An aliphatic eight-carbon chain (C3-C4-C5-C6-C7-C8-C9-C10) was detected on the basis of ^1^H-^1^H COSY experiment. HMBC correlations from H-14 to C-13, C-15, C-12 and C-16, from H-12 to C-13, C-14 and C-16, from H_3_-17 to C-15 indicated the presence of a tetrasubstituted benzene ring and two aromatic carbons were oxygenated, consistent with the chemical shifts pattern of the aromatic carbons and coupling constants of aromatic protons (Figure 2). The ester carbonyl must attach to C-16, and ester oxygen and C-11 was linked to C-3, C-10 respectively to form a dodecane-membered ring, satisfying the unsaturation degrees of the molecular formula. This connectivity was further confirmed by the distinct HMBC crosspeaks of H-10 with C-11, C-12 and C-16, of H-3 with C-1. The remaining two hydroxyl groups were bonded to C-5 and C-6 respectively to complete the planar structure of the compound **3**. 

Firstly, we tried to obtain some information about the absolute configuration of (5*S*,6*S*)-dihydroxylasiodiplodin by CD spectroscopy due to the small quantity available. Careful inspection of the eight computed ECD curves resulting from the three chiral centers of the compound **3**, showed that the experimental CD spectrum matched well with the ECD curves of the 3*S*-stereochemistry (Figure S38). Actually, the ECD curves of four configuration options of the 3*S* stereochemistry were identical, which indicated the different configurations at C-5 and C-6 positons didn’t affect the ECD absorbance. From the biosynthetic standpoint, the absolute stereochemistry of chiral centers C-3 and C-5 of compound **3** should be consistent with that of the known compound (5*S*)-hydroxy-lasiodiplodin [5]. For the 12-membered ring lactone 5-hydroxylasiodiplodin, the carbon chemical shift of 5*S* at δ_C_ 66.7 and 5*R* at δ_C_ 70.6 in CDCl_3_ solvent were distinctly different. The chemical shift at the C-5 position of compound **3** was at δ_C_ 64.5 in DMSO-*d_6_* and δ_C_ 67.1 in CD_3_OD, respectively. Apart from the solvent effect, the chemical shift at C-5 of compound **3** was consistent with that of (5*S*)-hydroxylasiodiplodin. Correspondingly, the stereochemistry of C-3 and C-5 of compound **3** was determined as 3*S*, 5*S*. For the other analogue (*3S*),(*6R*)-6-hydroxylasiodiplodin [6], the carbon chemical shift of *6R* at δ_C_ 70.5 in CDCl_3_/CD_3_OD solvent was distinctly different from the carbon chemical shift at C-6 position of compound **3** at δ_C_ 76.3 in CD_3_OD solvent. Furthermore, the NOESY spectrum of compound **3** displayed a NOE correlation between H-5 and H-6, therefore, the absolute configuration of (5*S*,6*S*)-dihydroxylasiodiplodin was proposed to be (3*S*,5*S*,6*S*).

## 3. Experimental Section 

### 3.1. General Procedures

Preparative HPLC was performed using a Shimadzu LC-20AT HPLC pump (Shimadzu Corporation, Kyoto, Japan) equipped with an SPD-20A dual λ absorbance detector (Shimadzu Corporation) and a Shim-pack PRC-ODS HPLC column (250 mm × 20 mm, Shimadzu Corporation). Column chromatography made use of silica gel (200~300 mesh, Marine Chemical Factory, Qingdao, China), Sephadex LH-20 (40–70 μm, greenherbs, Beijing, China). Optical rotations were measured using a Schmidt and Haensch Polartronic HNQW5 optical rotation spectrometer (SCHMIDT + HAENSCH GmbH & Co., Berlin, Germany). CD spectra were measured on a JASCO J-810 circular dichroism spectrometer (JASCO International Co. Ltd., Tokyo, Japan). UV spectra were recorded on a Shimadzu UV-Vis-NIR spectrophotometer (Shimadzu Corporation). IR spectra were acquired on a PerkinElmer Frontier Fourier transform infrared (FT-IR) spectrophotometer (Perkin Elmer Inc., Waltham, MA, USA) with an Ever-Glo mid/near-IR source. 1D and 2D NMR spectra were recorded on a Bruker Avance II 400 spectrometer (Bruker BioSpin AG, Fällanden, Switzerland). The chemical shifts are relative to the residual solvent signals (DMSO-*d*_6_: δ_H_ 2.50 and δ_C_ 39.52; CD_3_OD: δ_H_ 3.31 and δ_C_ 49.00; acetone-*d*_6_: δ_H_ 2.05 and δ_C_ 29.84). Mass spectra were obtained on a Thermo DSQ EI low-resolution mass spectrometer (Thermo Fisher Scientific, Waltham, MA, USA) and a Thermo MAT95XP EI high-resolution mass spectrometer (Thermo Fisher Scientific). ESI-MS spectra were measured on a Thermo Finnigan LCQ DECA XP LC/MS machine (Thermo Fisher Scientific, Waltham, MA, USA) with an ESI probe operating in the positive-ion mode.

### 3.2. Fungal Material

The marine fungus *Pseudallescheria ellipsoidea* F42-3 was isolated from the inner tissue of the soft coral *Lobophytum crassum* collected from Hainan Sanya National Coral Reef Reserve, China. This fungal strain was maintained in 15% glycerol aqueous solution at −80 °C. A voucher specimen was deposited in the School of Pharmaceutical Sciences, Sun Yat-sen University, Guangzhou, China. Analysis of the ITS rDNA by BLAST database screening provided a 99.7% match to *Pseudallescheria ellipsoidea* (compared with JQ690937).

### 3.3. Extraction, Isolation and Characterization

The fermentation medium was glucose 10 g/L, peptone 5 g/L, yeast extract 2 g/L, sea water 1 L and pH 7.5 (GPY medium). Fungal mycelia were cut and transferred aseptically to 500-mL Erlenmeyer flasks, each containing 200 mL sterilized GPY liquid medium. The flasks were incubated at 28 °C on a rotary shaker (120 rpm) for 20 days. Sixty liters of liquid culture were filtered through cheesecloth. The culture broth was extracted three times with EtOAc and then was concentrated under reduced pressure to afford an extract (8.9 g). The extract was subjected to column chromatography over silica gel eluting with a gradient of petroleum ether–EtOAc (100:0–0:100, *v*/*v*) followed by EtOAc–MeOH (100:0–0:100) to yield 10 fractions (Fr.1–Fr.10). Fr.5 was separated by Sephadex LH-20 column to give five fractions (Fr.5.1–Fr.5.5). The fraction Fr.5.4 was recrystallized from MeOH to furnish **1** (2.5 mg). Fr.5.2 was further separated via reversed-phase semi-preparative HPLC eluting with MeCN–H_2_O (50:50, *v*/*v*) to obtain **2** (1.2 mg). Fr. 4 was initially separated by Sephadex LH-20 column to yield 7 fractions (Fr.4.1–Fr.4.7). Fr.4.2 and Fr.4.5 was further isolated by reversed-phase semi-preparative HPLC eluting with MeOH–H_2_O (50:50; 60:40 respectively, *v*/*v*) to yield **3** (2.1 mg) and **4** (2.3 mg).

*Pseudellone D* (**1**): pale yellow solid; ${\left[\alpha \right]}_{D}^{20}$ +6.01 (*c* 0.3, MeOH); UV (MeOH) λ_max_ (log ε) 213 nm (3.82), 278 nm (3.19); CD (MeCN): 221 (Δε+2.18), 241 (Δε−2.48); IR υ_max_ 3406, 3210, 2928, 1667, 1423, 1237, 1120, 747 cm^−1^; ^1^H- and ^13^C-NMR data, see Table 1; HREIMS *m*/*z* 303.1029 [M]^+^ (calcd for C_15_H_17_N_3_O_2_S, 303.1041).

*Lasiodipline F* (**2**): white solid; CD (MeCN): 204 (Δε−5.62), 220 (Δε+2.25); ^1^H- and ^13^C-NMR data, see Supplementary Figures S11 and S12; LRESIMS *m*/*z* 301, 300, 265, 187, 125.

(5*S*, 6*S*)-*dihydroxylasiodiplodin* (**3**): white solid; UV (MeOH) λ_max_ (log ε) 203 nm (3.72), 280 nm (3.60); CD (MeCN): 208 (Δε+10.42), 247 (Δε−8.87); IR υ_max_ 2927, 2854, 2360, 1699, 1603, 1463, 1270, 1163, 1095 cm^−1^; ^1^H- and ^13^C-NMR data, see Table 1; HREIMS *m*/*z* 324.1562 [M]^+^ (calcd for C_17_H_24_O_6_, 324.1573).

*(5S)-hydroxylasiodiplodin* (**4**): white solid; CD (MeCN): 208 (Δε+18.50), 245 (Δε−11.48); ^1^H- and ^13^C-NMR data, see Supplementary Figures S30 and S31; LRESIMS *m*/*z* 310, 309, 191, 169, 148, 107, 85.

## 4. Conclusions

In conclusion, two additional new compounds including one sulfur-containing diketopiperazine, pseudellone D (**1**), and one twelve-membered lactone lasiodiplodin derivative, (5*S*,6*S*)-dihydroxylasiodiplodin (**3**), together with the two known compounds lasiodipline F (**2**), and (5*S*)-hydroxylasiodiplodin (**4**) were isolated from the marine-derived fungus *Pseudallescheria ellipsoidea* F42-3 associated with the soft coral *Lobophytum crassum*. For lasiodipline F (**2**), it has been discovered from the culture of *Lasiodiplodia pseudotheobromae* F2 and only been reported once as a novel natural compound [4]. The diketopiperazines pseudellone D (**1**) and lasiodipline F (**2**) contained a rare monomethylthio group. For sulfur-containing diketopiperazines, commonly two methylthio groups are substituted at α-carbon positions on the diketopiperazine ring, and monomethylthio-containing compounds are rarely discovered in Nature. 

## Figures and Tables

**Table 1:** ^1^H- and ^13^C-NMR Data (400 and 100 MHz, resp. δ in ppm) of compounds **1** and **3**.

No.	1 (acetone-*d*_6_)		3 (CD_3_OD)	
	δ_H_ (*J* in Hz)	δ_C_	δ_H_ (*J* in Hz)	δ_C_
1		166.5, C		170.5, C
2	3.44, qd (7.2, 2.4)	51.9, CH		
3	7.17 (brs) ^a^		5.24, m	71.1, CH
4		169.3, C	2.28, ddd (15.6, 6.4, 34.0); 1.72, dt (15.2, 5.2)	37.6, CH_2_
5		68.1, C	3.94, td (6.0, 1.2)	70.5, CH
6	10.18 (brs) ^a^		3.68, tt (6.8, 1.6)	76.3, CH
7	3.78, d (14.4); 3.21, d (14.0)	35.3, CH_2_	1.67, m; 1.54, m	31.7, CH_2_
8		109.4, C	1.64, m; 1.35, m	23.1, CH_2_
9		129.0, C	2.43, dt (13.6, 5.2); 1.30, m	30.2, CH_2_
10	7.76, d (8.0)	120.2, CH	2.68, ddd (13.2, 10.4, 5.2); 1.20, m	30.2, CH_2_
11	6.99, td (7.2, 1.2)	119.8, CH		143.6, C
12	7.07, td (7.2, 1.2)	122.1, CH	6.25, d (2.0)	109.1, CH
13	7.35, d (8.4)	112.0, CH		160.9, C
14		137.2, C	6.29, d (2.0)	97.9, CH
15	7.50 (brs) ^a^	NH		159.4, C
16	7.24, d (2.4)	126.2, CH		117.8, C
17	1.35, d (6.8)	13.7, CH_3_	3.76, s	56.3, CH_3_
18	2.29, s	20.7, CH_3_	1.38, d (6.4)	19.5, CH_3_

^a^ These data may be interchanged.

## Supplementary Materials

The MS, NMR (1D and 2D) spectra of compounds **1**–**4** are available as supporting information. Supplementary materials can be accessed at: http://www.mdpi.com/1420-3049/21/4/442/s1.
