# Supplementary material for: Two Additional New Compounds from the Marine-Derived Fungus Pseudallescheria ellipsoidea F42-3
Source: Molecules. 2016 Apr 1;21(4):442. doi: 10.3390/molecules21040442 (PMC6274254; doi:10.3390/molecules21040442)
Supplement: Supplementary file 1 [file molecules-21-00442-s001.pdf]

Supplementary Materials: Additional Two New Compounds from the Marine-Derived Fungus *Pseudallescheria ellipsoidea* F42-3

Kun-Teng Wang [1], Meng-Yang Xu [2], Wei Liu[1], Hou-Jin Li [3], Jun Xu [2], De-Po Yang [2,4], Wen-Jian Lan [2,4] and Lai-You Wang [1,*]

List of Supporting Information

Figure S1. HR-EI mass spectrum of compound **1**.

Figure S2. ^{1}H-NMR spectrum of compound **1** in acetone-d_6 (400 MHz).

Figure S3. ^{13}C-NMR spectrum of compound **1** in acetone-d_6 (100 MHz).

Figure S4. DEPT 135 spectrum of compound **1** in acetone-d_6 (100 MHz).

Figure S5. DEPT 90 spectrum of compound **1** in acetone-d_6 (100 MHz).

Figure S6. HMQC spectrum of compound **1** in acetone-d_6.

Figure S7. ^{1}H-^{1}H COSY spectrum of compound **1** in acetone-d_6.

Figure S8. HMBC spectrum of compound **1** in acetone-d_6.

Figure S9. NOESY spectrum of compound **1** in acetone-d_6.

Figure S10. LR-ESI mass spectrum of compound **2**.

Figure S11. ^{1}H-NMR spectrum of compound **2** in DMSO-d_6 (400 MHz).

Figure S12. ^{13}C-NMR spectrum of compound **2** in DMSO-d_6 (100 MHz).

Figure S13. DEPT 135 spectrum of compound **2** in DMSO-d_6 (100 MHz).

Figure S14. DEPT 90 spectrum of compound **2** in DMSO-d_6 (100 MHz).

Figure S15. HMQC spectrum of compound **2** in DMSO-d_6.

Figure S16. ^{1}H-^{1}H COSY spectrum of compound **2** in DMSO-d_6.

Figure S17. HMBC spectrum of compound **2** in DMSO-d_6.

Figure S18. NOESY spectrum of compound **2** in DMSO-d_6.

Figure S19. HR-EI mass spectrum of compound **3**.

Figure S20. ^{1}H-NMR spectrum of compound **3** in CD_3OD (400 MHz).

Figure S21. ^{13}C-NMR spectrum of compound **3** in CD_3OD (100 MHz).

Figure S22. ^{13}C NMR spectrum of compound **3** in acetone-d_6 (100 MHz).

Figure S23. DEPT 135 spectrum of compound **3** in acetone-d_6 (100 MHz).

Figure S24. DEPT 90 spectrum of compound **3** in acetone-d_6 (100 MHz).

Figure S25. HMQC spectrum of compound **3** in CD_3OD.

Figure S26. ^{1}H-^{1}H COSY spectrum of compound **3** in CD_3OD.

Figure S27. HMBC spectrum of compound **3** in CD_3OD.

Figure S28. NOESY spectrum of compound **3** in CD_3OD.

Figure S29. LR-ESI mass spectrum of compound **4**.

Figure S30. ^{1}H-NMR spectrum of compound **4** in DMSO-d_6 (400 MHz).

Figure S31. ^{13}C-NMR spectrum of compound **4** in DMSO-d_6 (100 MHz).

Figure S32. DEPT 135 spectrum of compound **4** in DMSO-d_6 (100 MHz).

Figure S33. DEPT 90 spectrum of compound **4** in DMSO-d_6 (100 MHz).

Figure S34. HMQC spectrum of compound **4** in DMSO-d_6.

Figure S35. ^{1}H-^{1}H COSY spectrum of compound **4** in DMSO-d_6.

Figure S36. HMBC spectrum of compound **4** in DMSO-*d*6.

Figure S37. NOESY spectrum of compound **4** in DMSO-*d*6.

Figure S38. Comparison of the experimental ECD spectra of **3** with the calculated ECD spectra for four (3*S*) stereochemical options.

Figure S39. Experimental CD spectra of **2**.

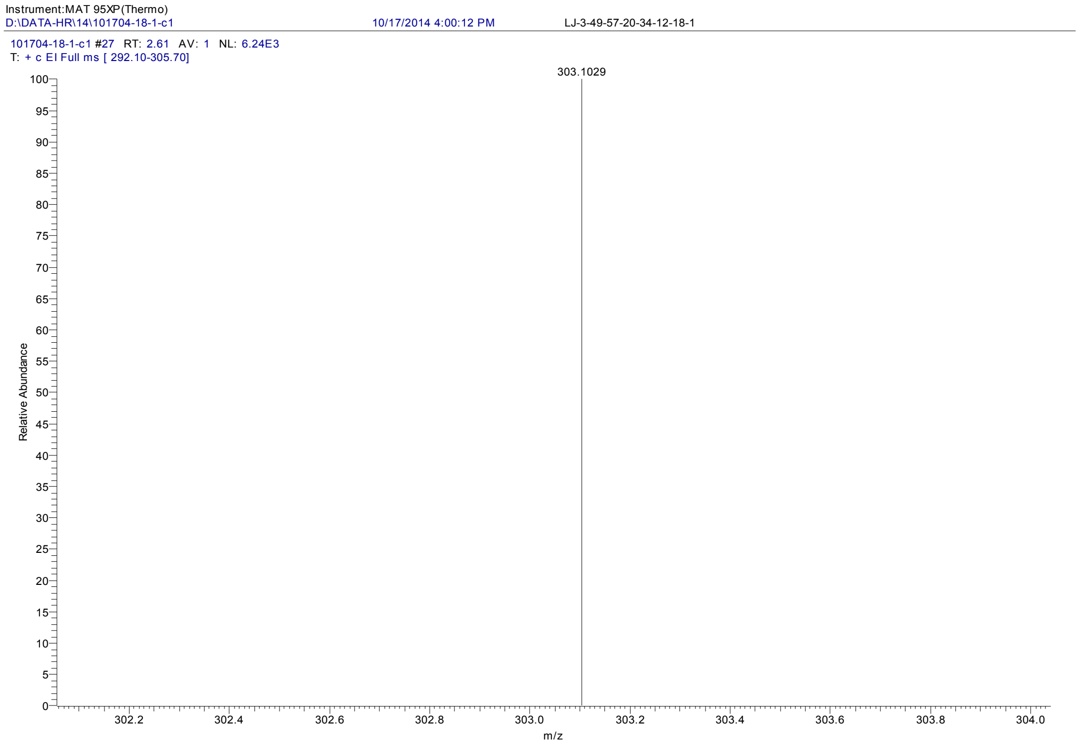

Figure S1. HR-EI mass spectrum of compound **1**.

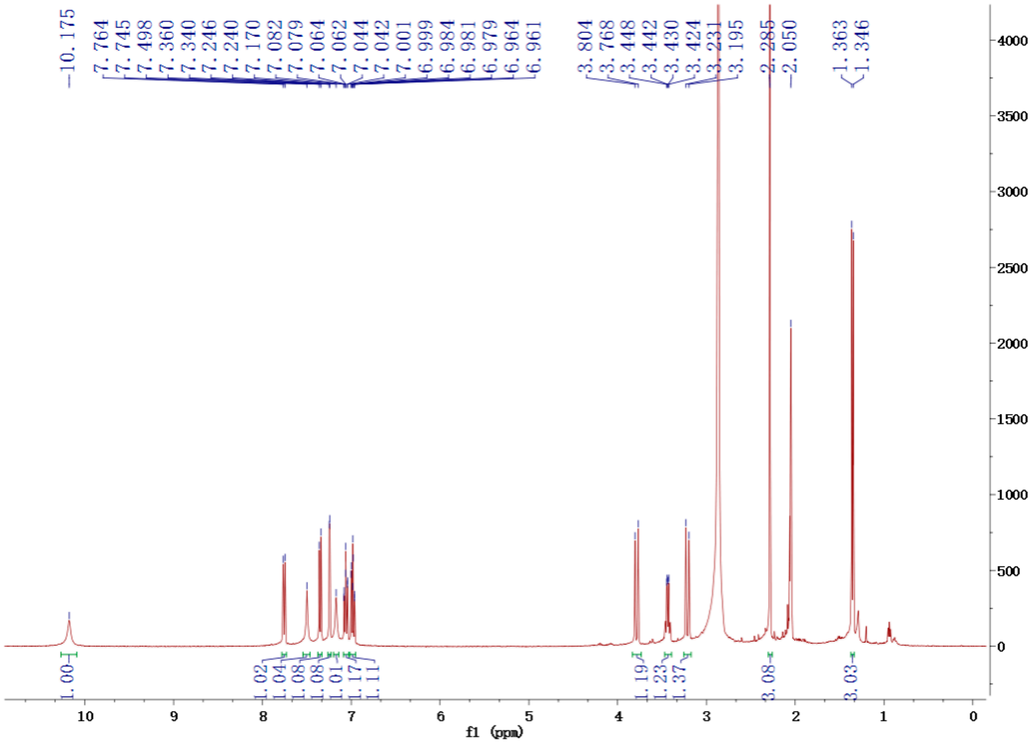

Figure S2. ^{1}H-NMR spectrum of compound **1** in acetone--*d*6 (400 MHz).

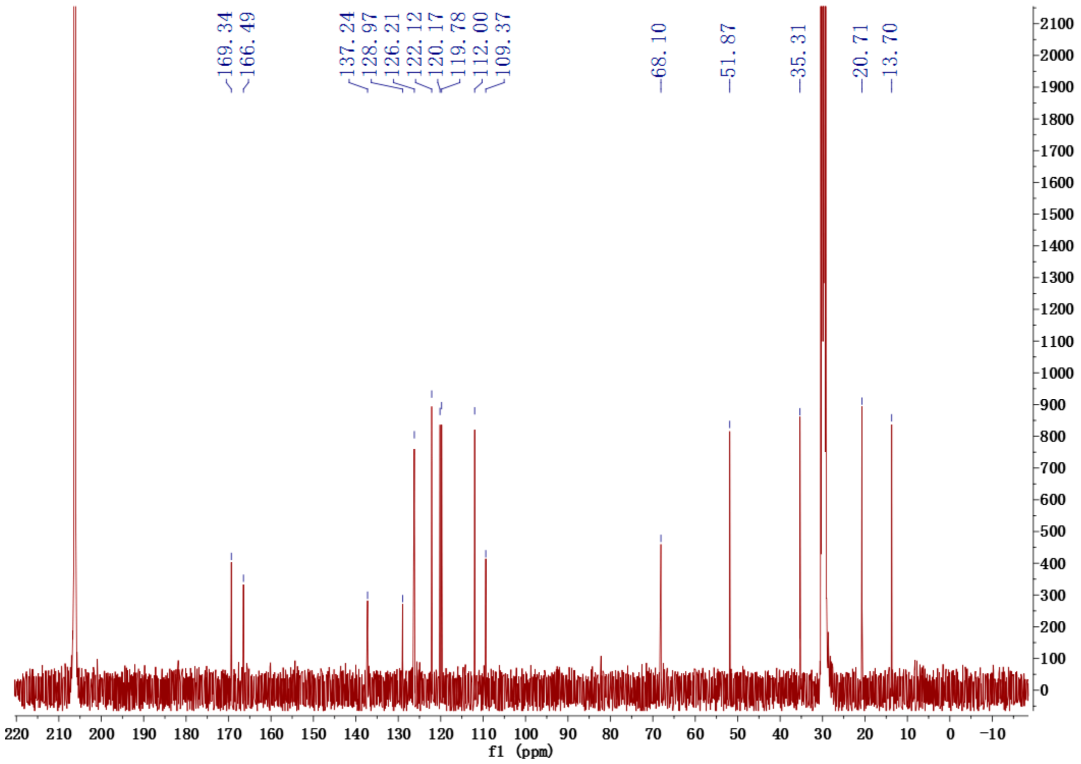

Figure S3. ^{13}C-NMR spectrum of compound **1** in acetone-d_6 (100 MHz).

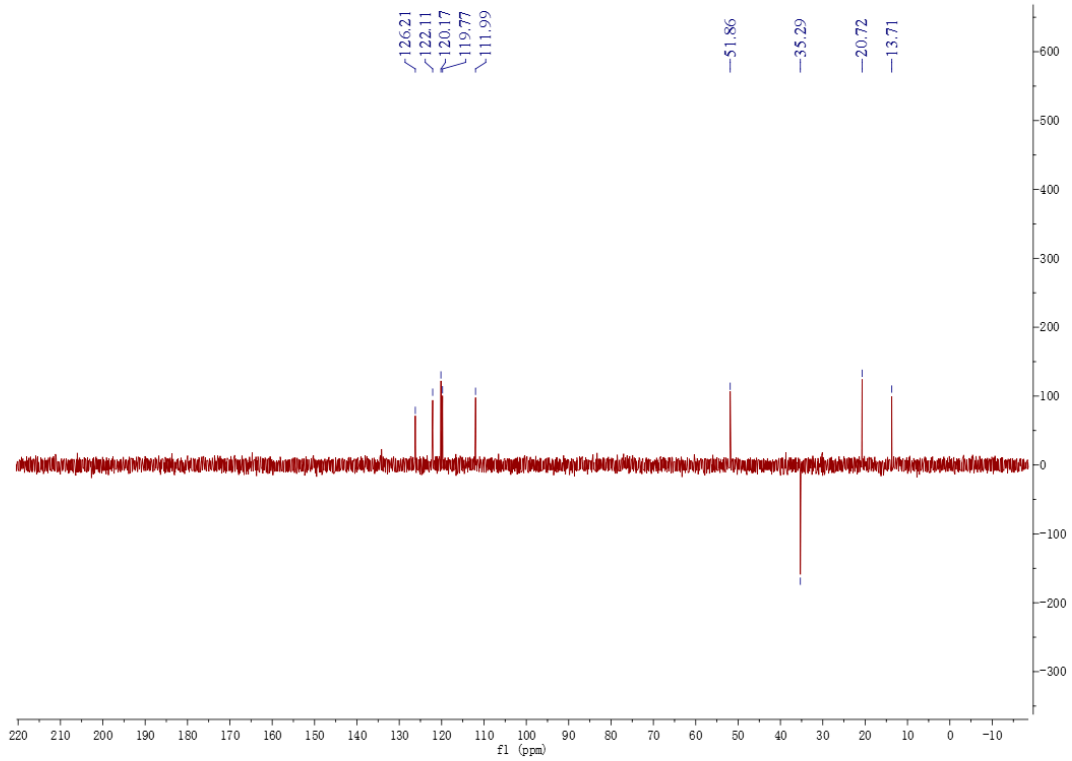

Figure S4. DEPT135 spectrum of compound **1** in acetone-d_6 (100 MHz).

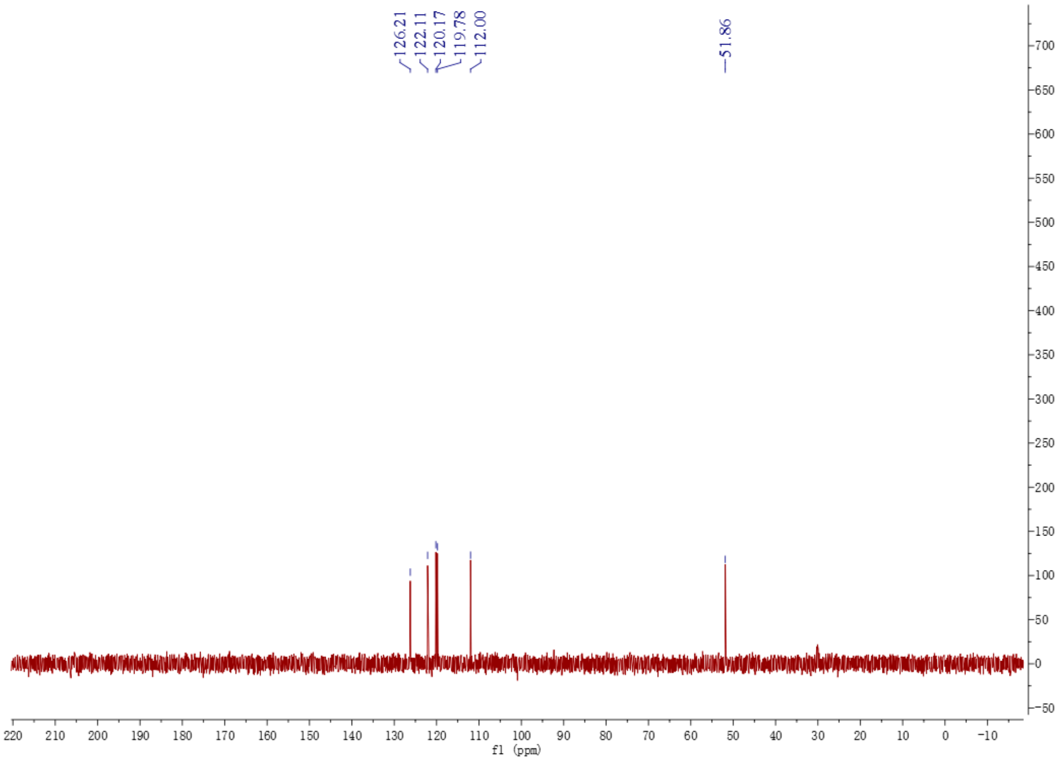

Figure S5. DEPT90 spectrum of compound **1** in acetone-d_6 (100 MHz).

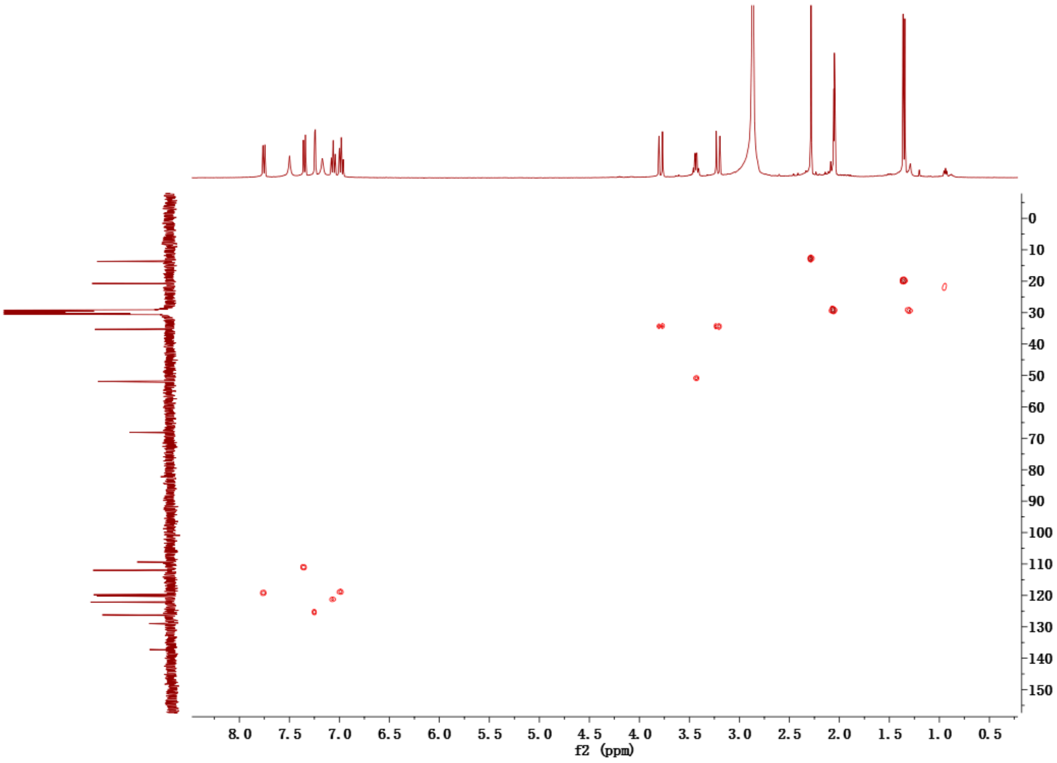

Figure S6. HMQC spectrum of compound **1** in acetone-d_6.

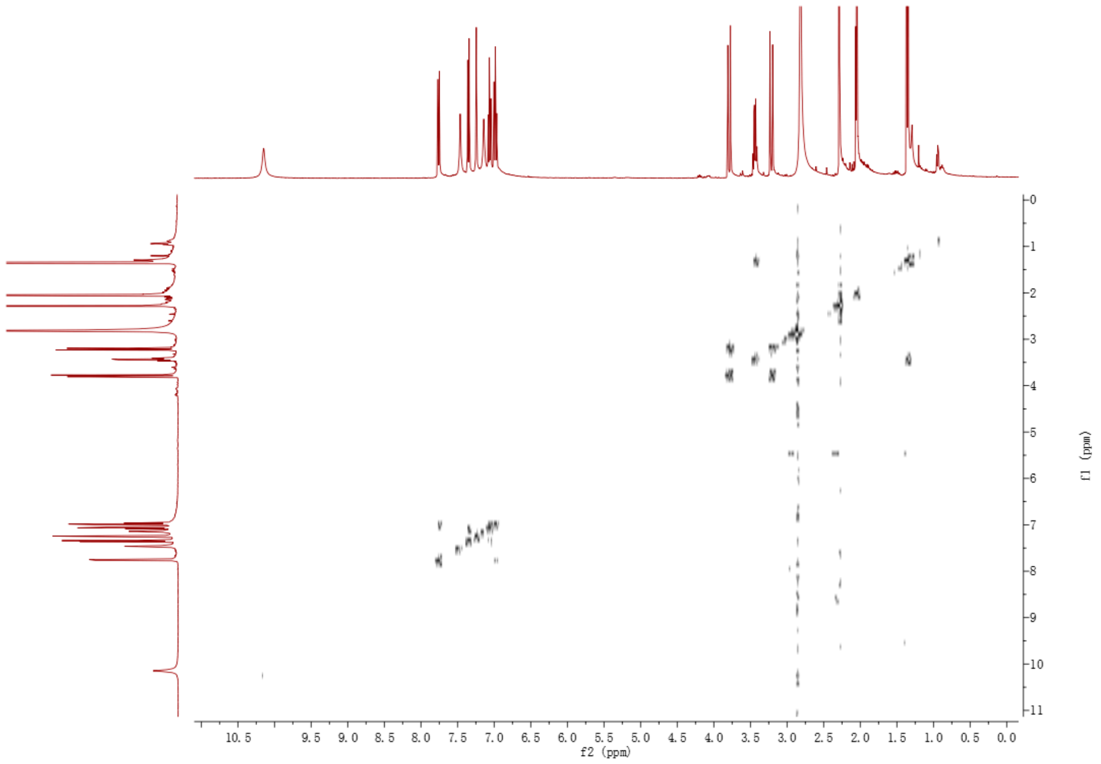

Figure S7. ^1H-^1H COSY spectrum of compound **1** in acetone-d_6.

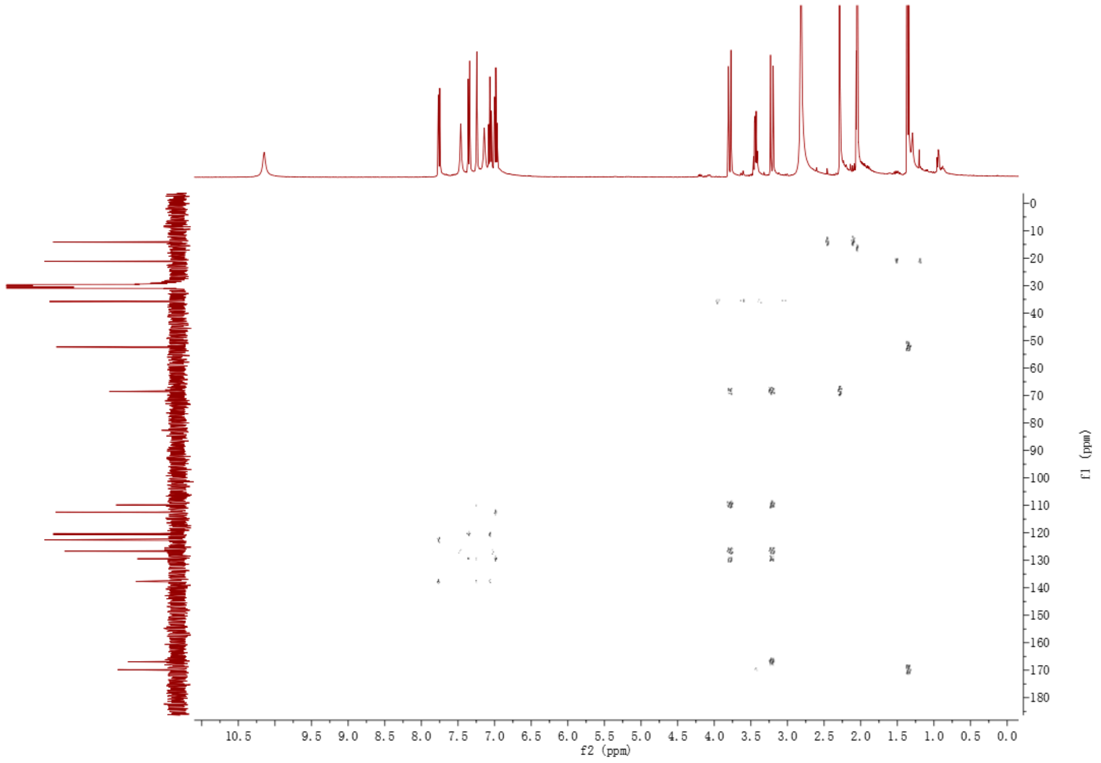

Figure S8. HMBC spectrum of compound **1** in acetone-d_6.

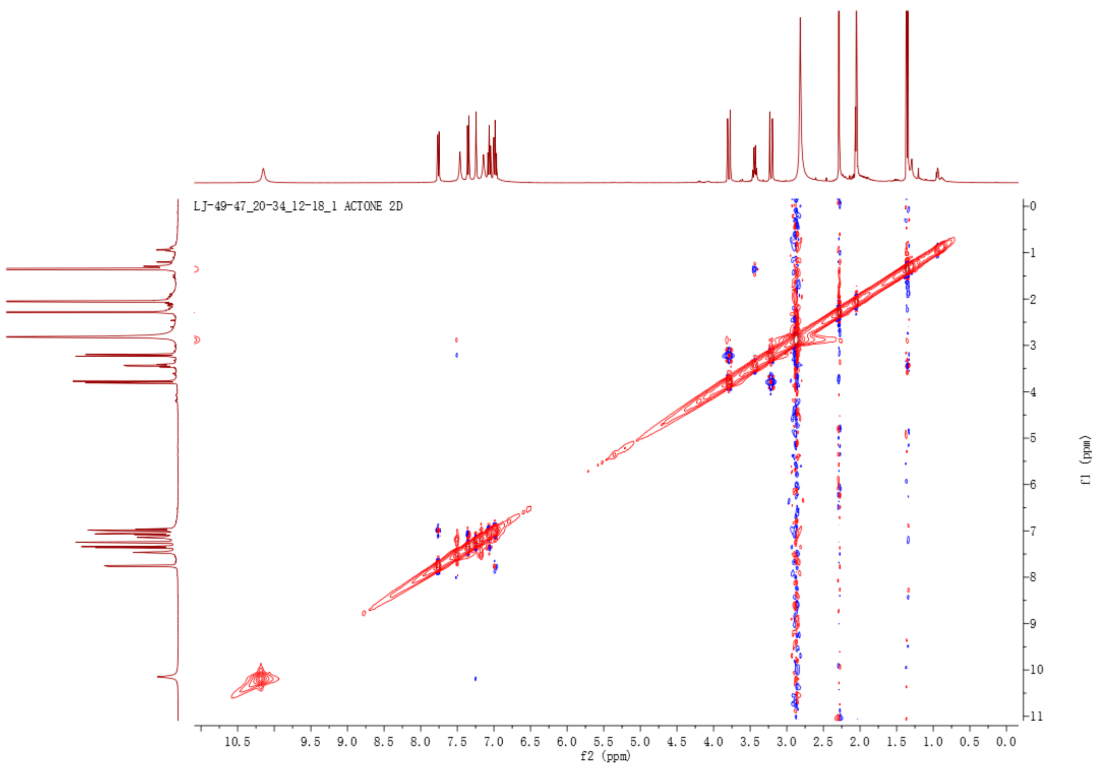

Figure S9. NOESY spectrum of compound **1** in acetone-d_6.

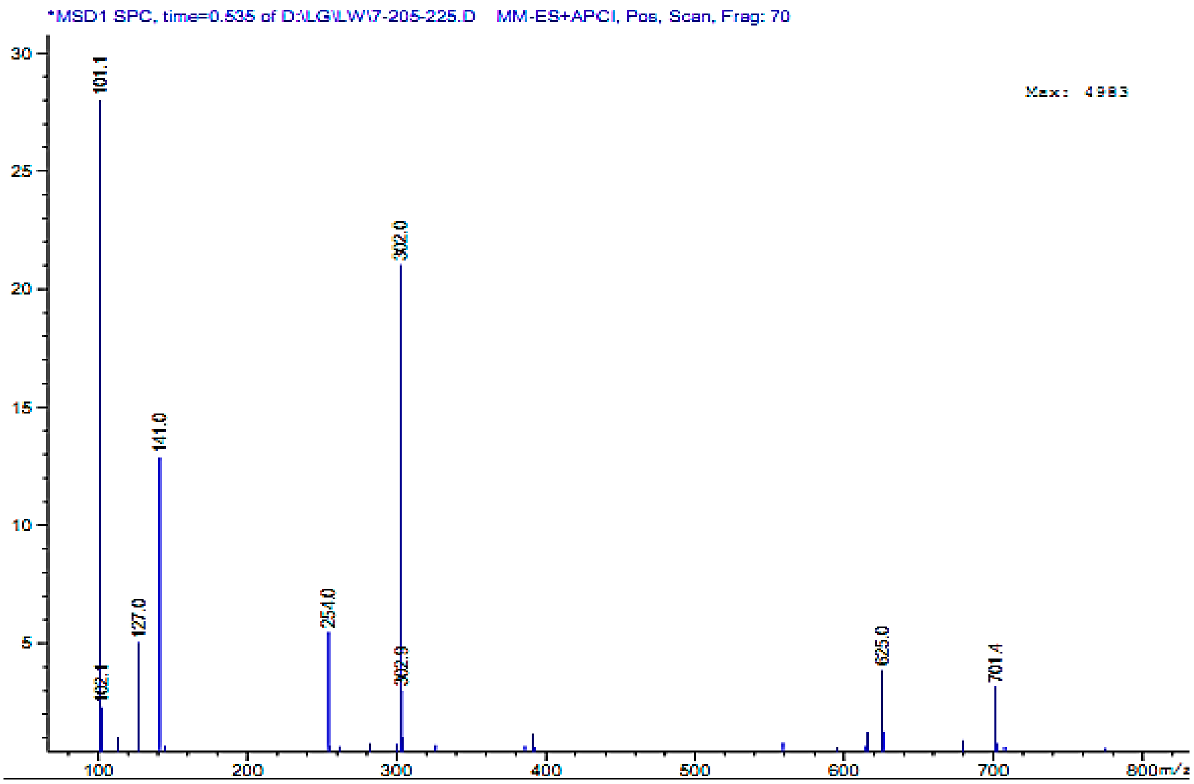

Figure S10. LR-ESI mass spectrum of compound **2**.

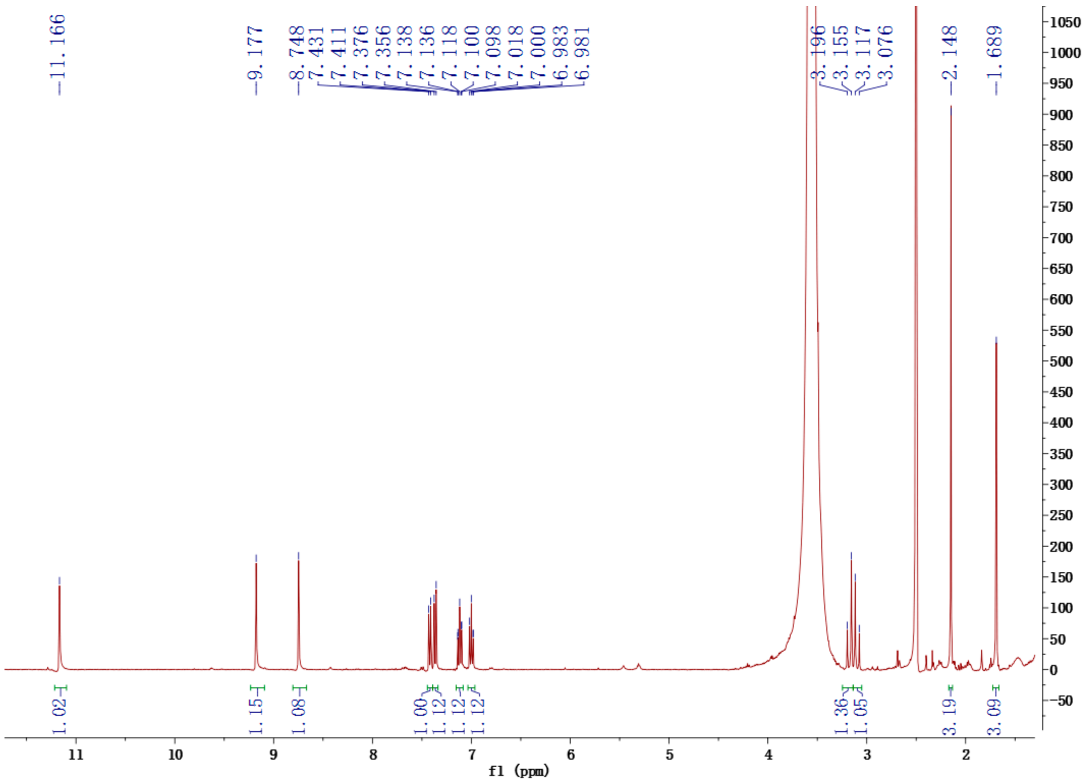

Figure S11. ^{1}H-NMR spectrum of compound **2** in DMSO-d_6 (400 MHz).

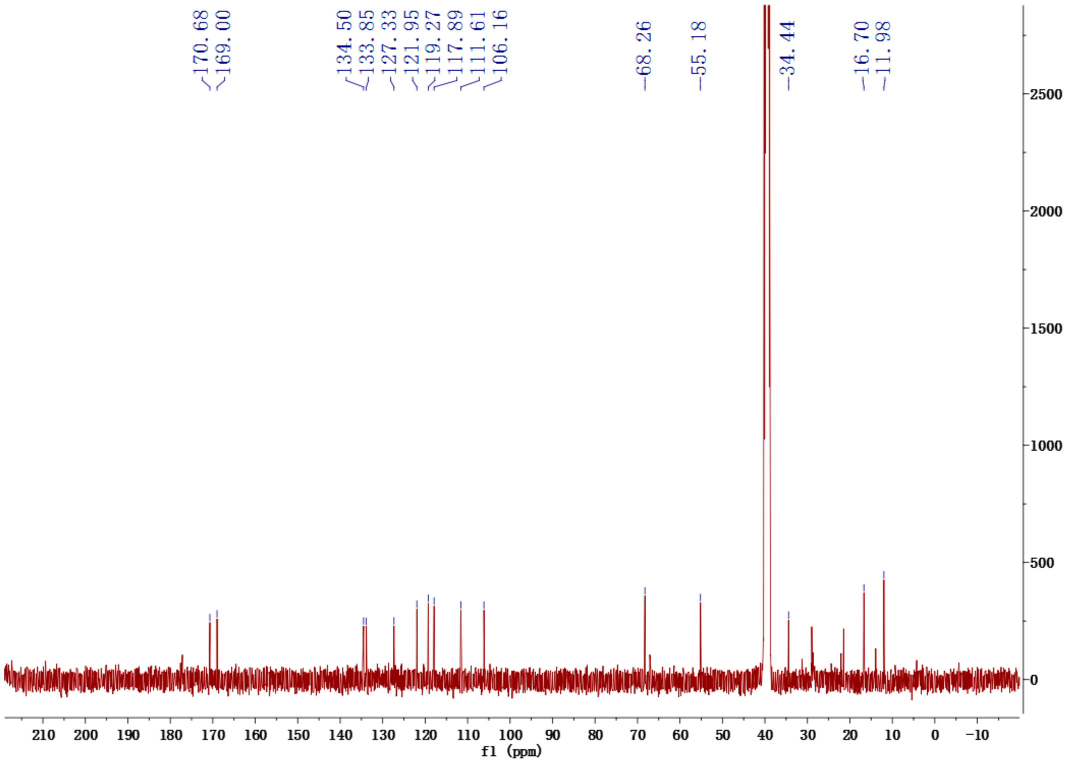

Figure S12. ^{13}C-NMR spectrum of compound **2** in DMSO-d_6 (100 MHz).

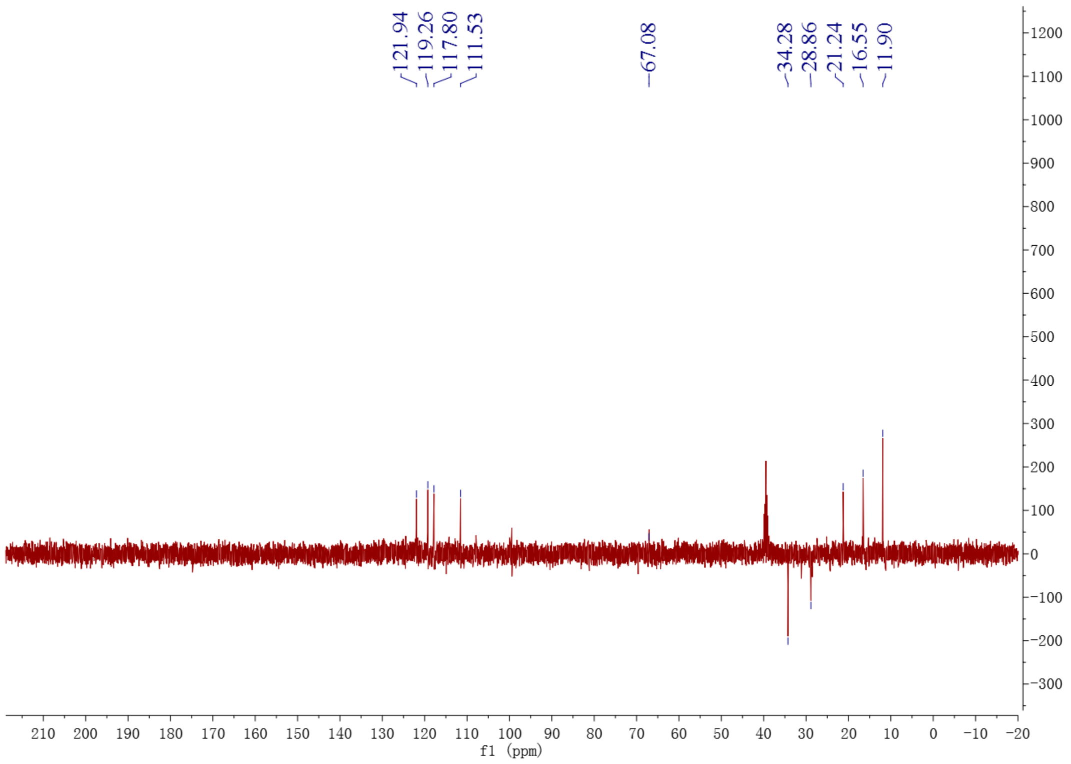

Figure S13. DEPT135 spectrum of compound **2** in DMSO-d_6 (100 MHz).

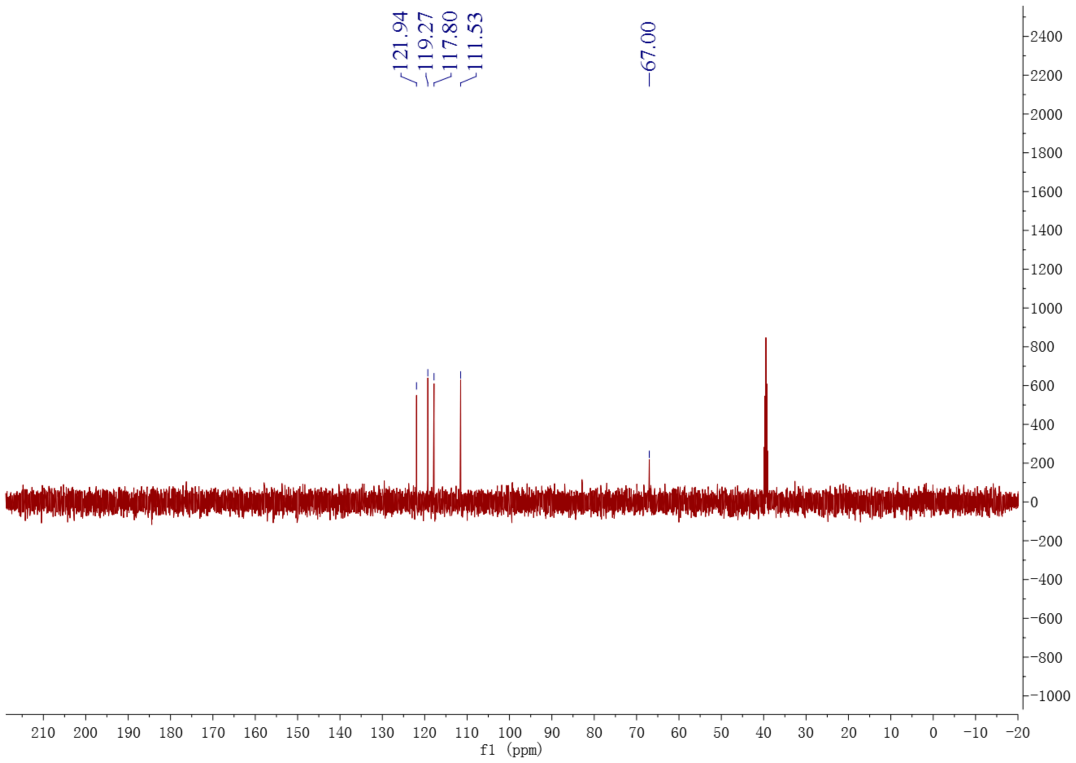

Figure S14. DEPT90 spectrum of compound **2** in DMSO-d_6 (100 MHz).

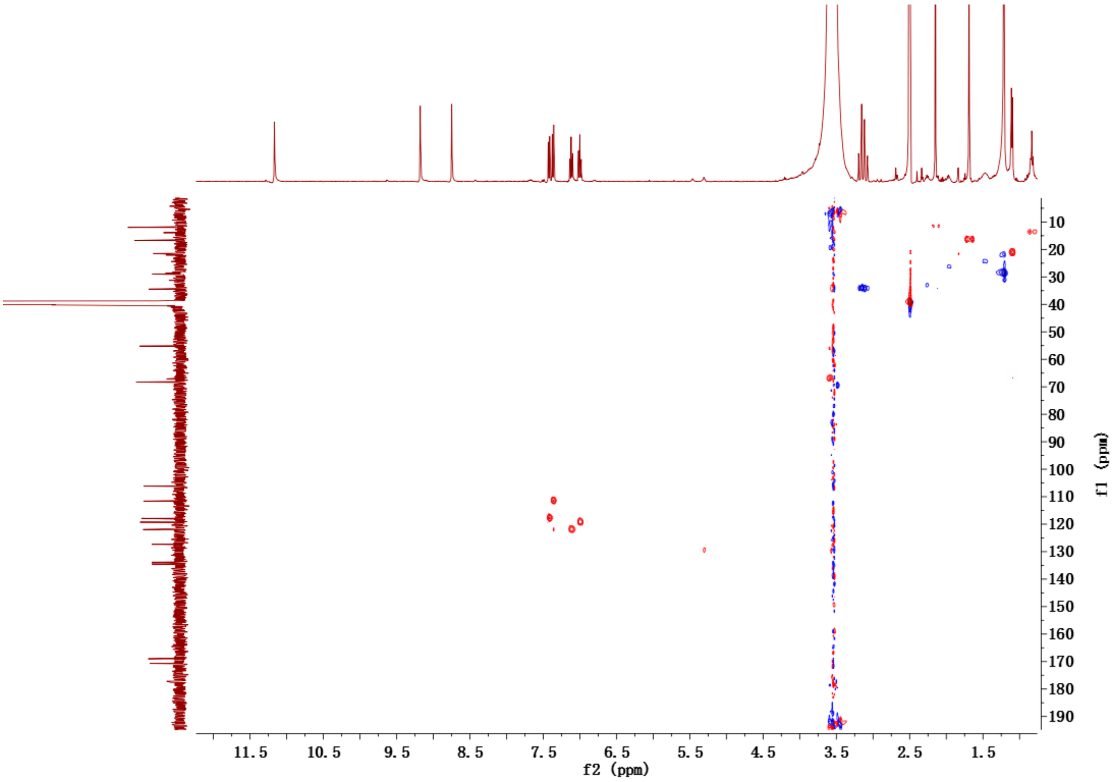

Figure S15. HMQC spectrum of compound **2** in DMSO-*d*6.

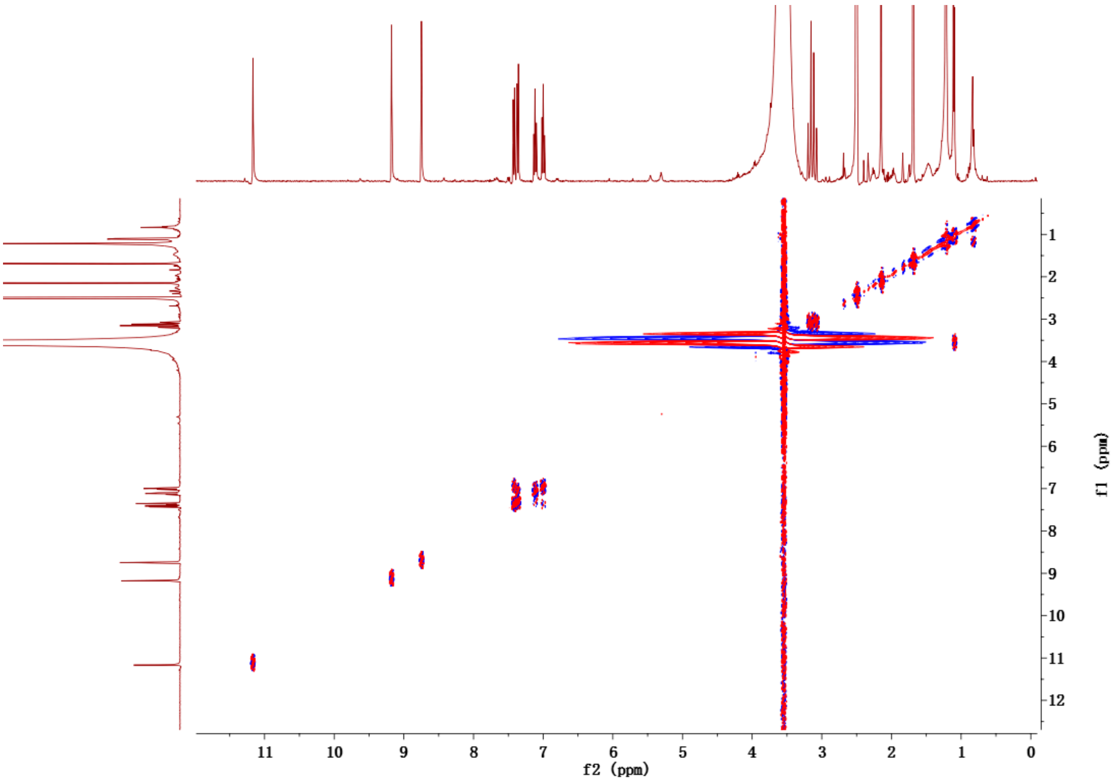

Figure S16. ^{1}H-^{1}HCOSY spectrum of compound **2** in DMSO-*d*6.

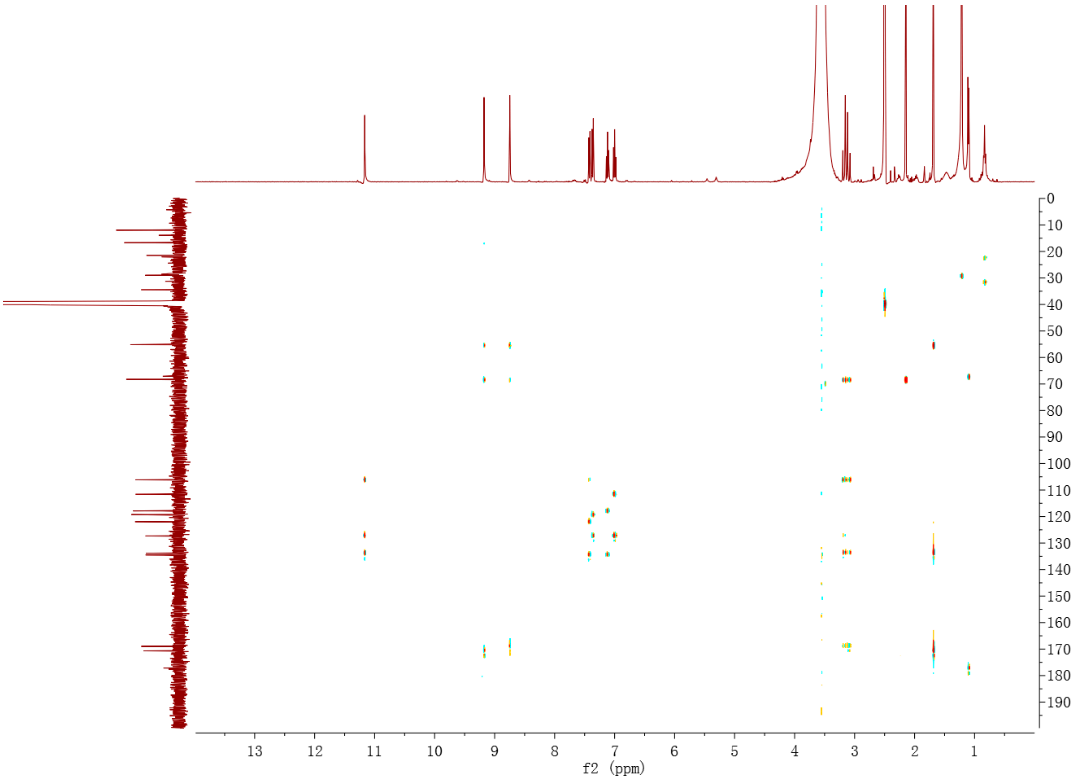

Figure S17. HMBC spectrum of compound **2** in DMSO-d_6.

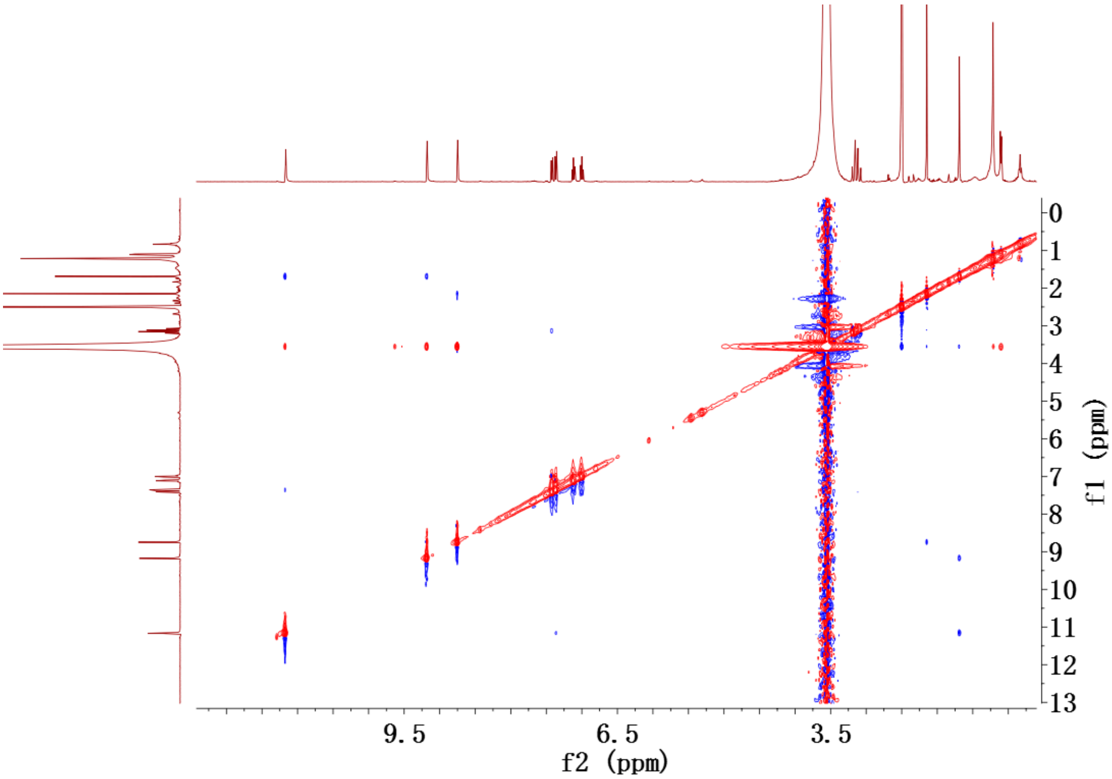

Figure S18. NOESY spectrum of compound **2** in DMSO-d_6.

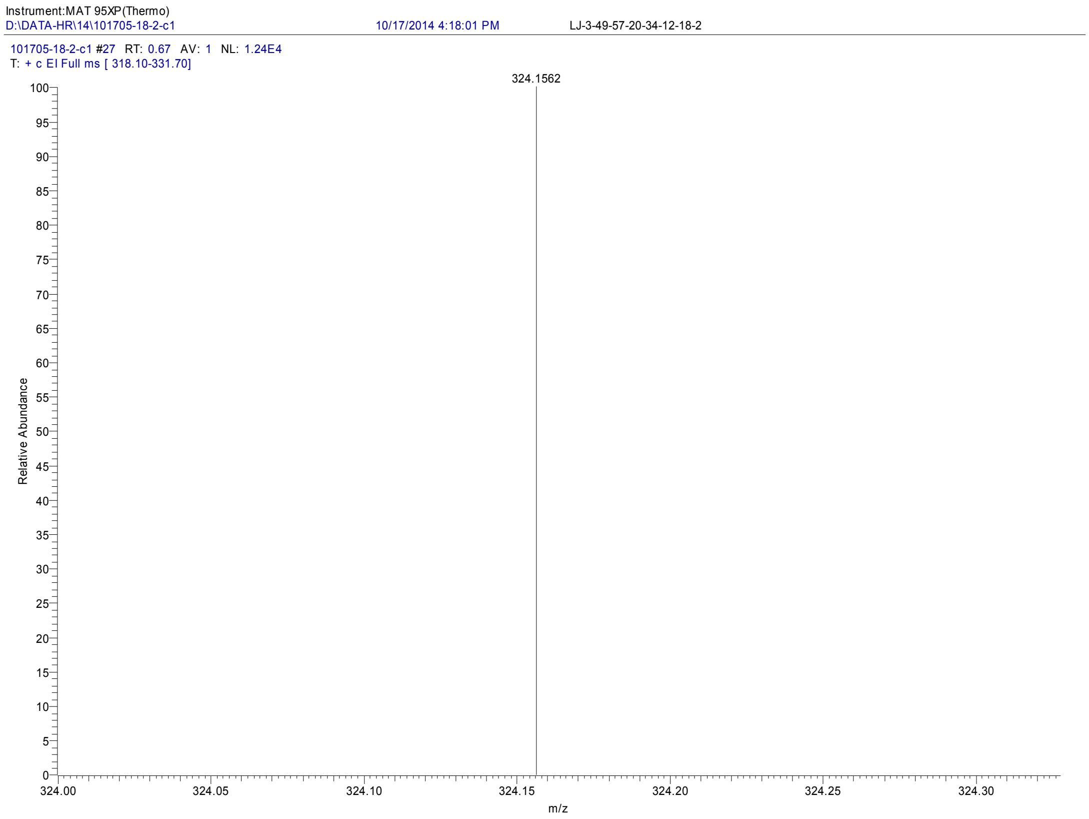

Figure S19. HREI Mass spectrum of compound **3**.

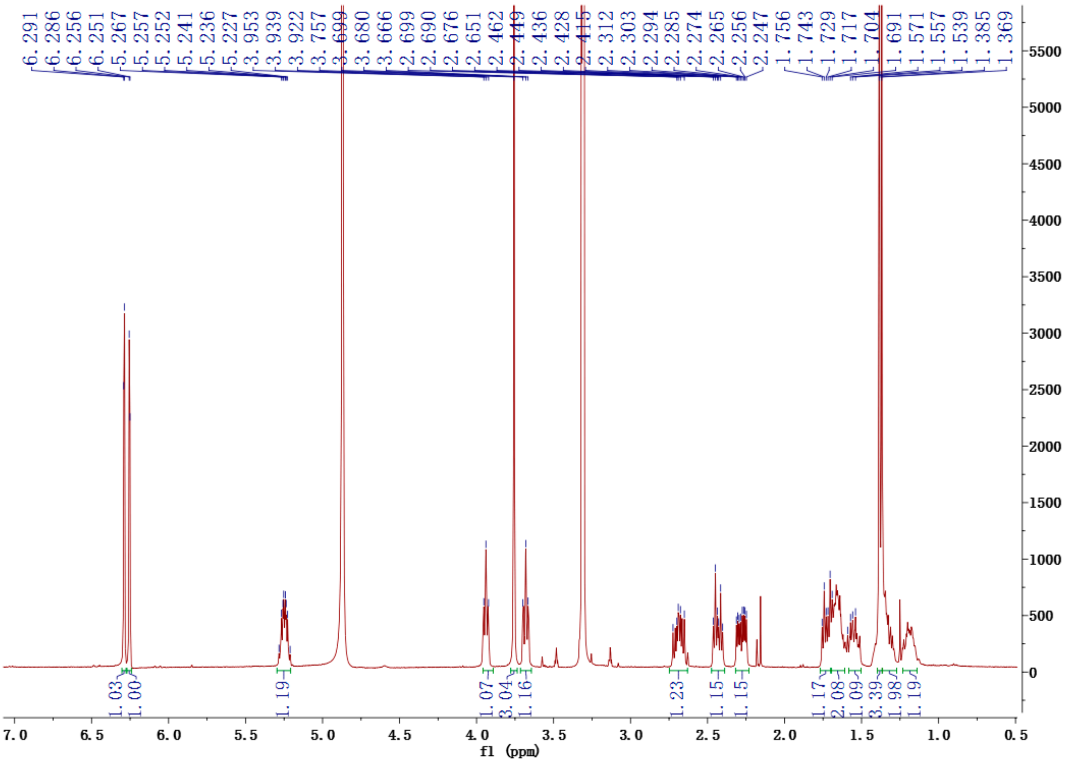

Figure S20. ^{1}H-NMR spectrum of compound **3** in CD_3OD (400 MHz).

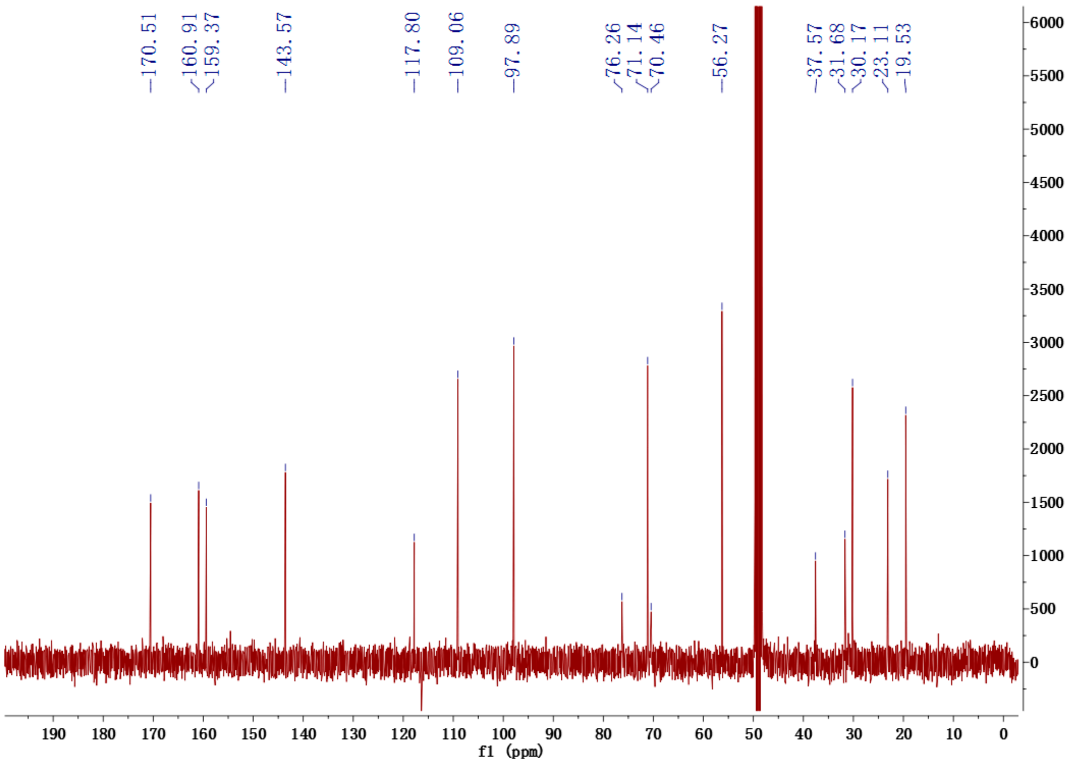

Figure S21. ^{13}C-NMR spectrum of compound **3** in CD_3OD (100 MHz).

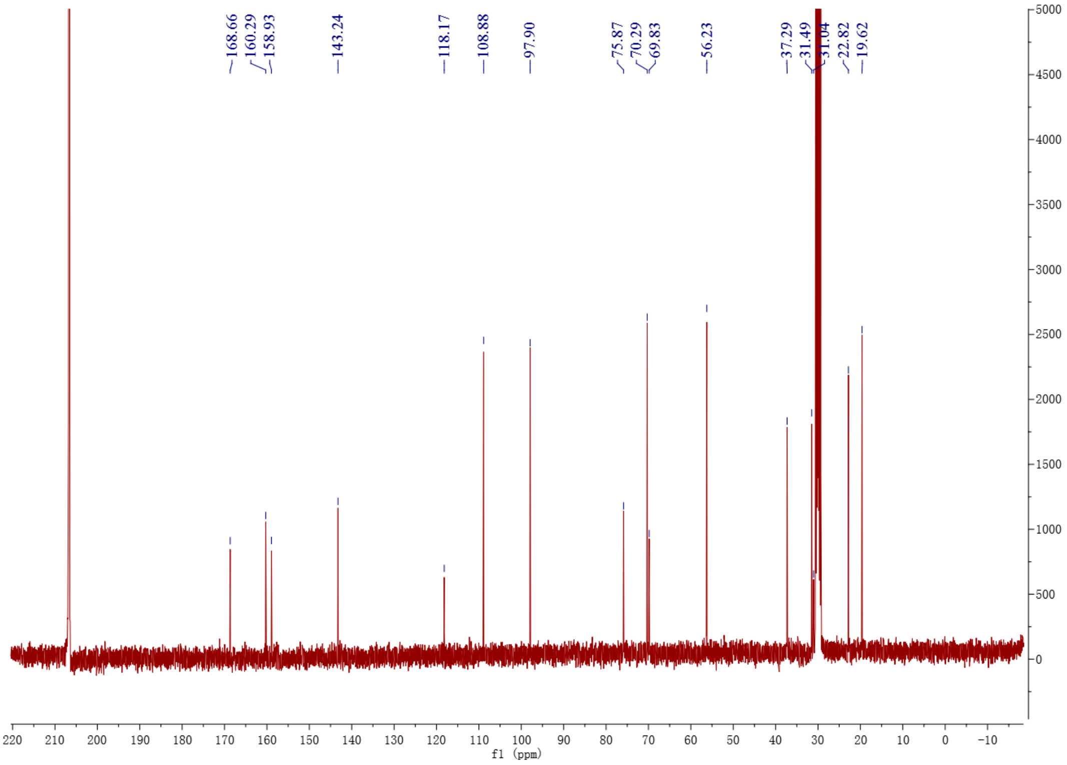

Figure S22. ^{13}C-NMR spectrum of compound **3** in acetone-d_6 (100 MHz).

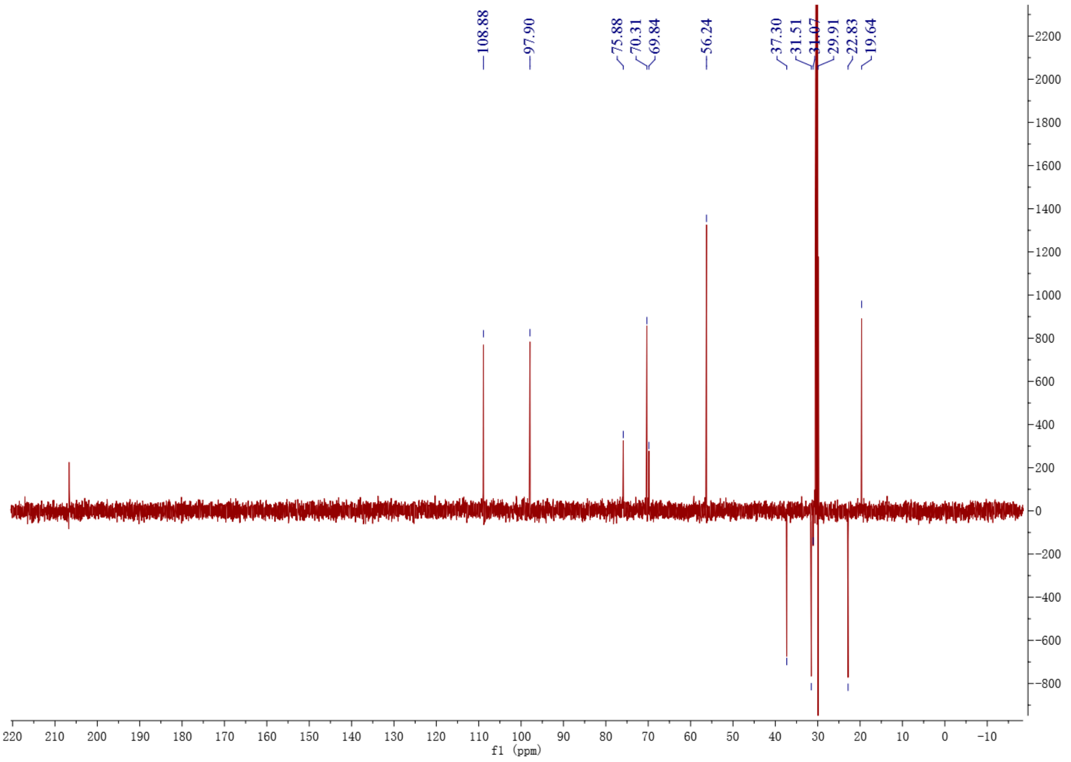

Figure S23. DEPT135 spectrum of compound **3** in acetone-d_6 (100 MHz).

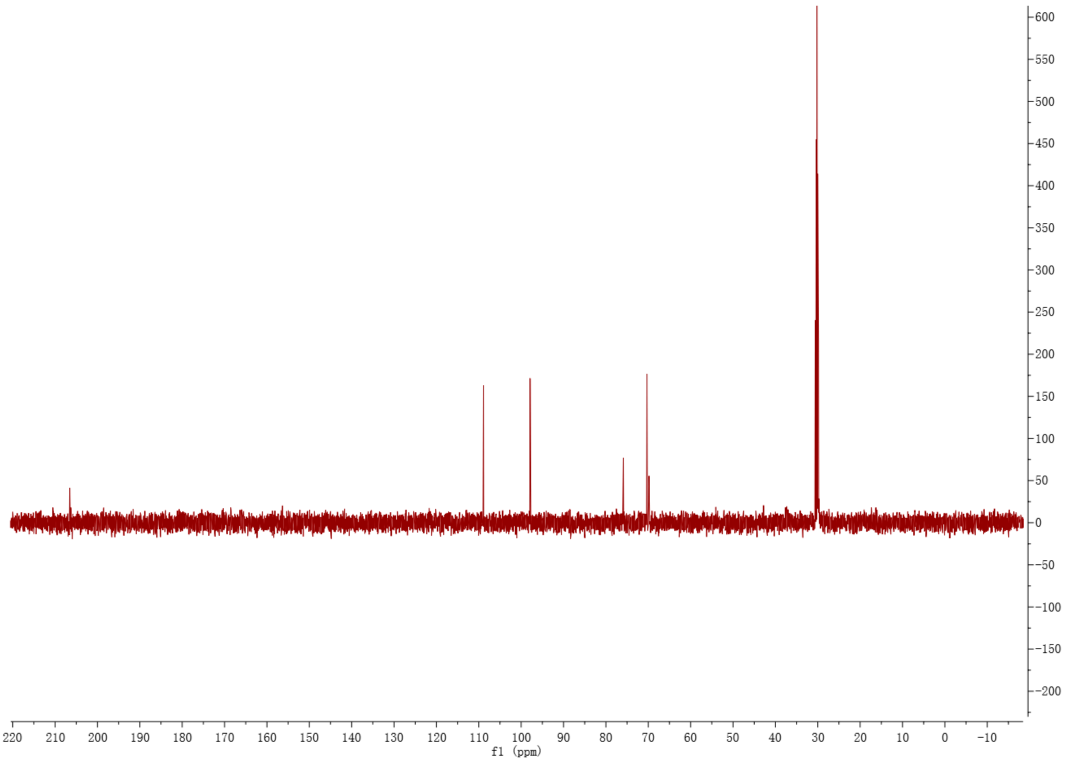

Figure S24. DEPT90 spectrum of compound **6** in acetone-d_6 (100 MHz).

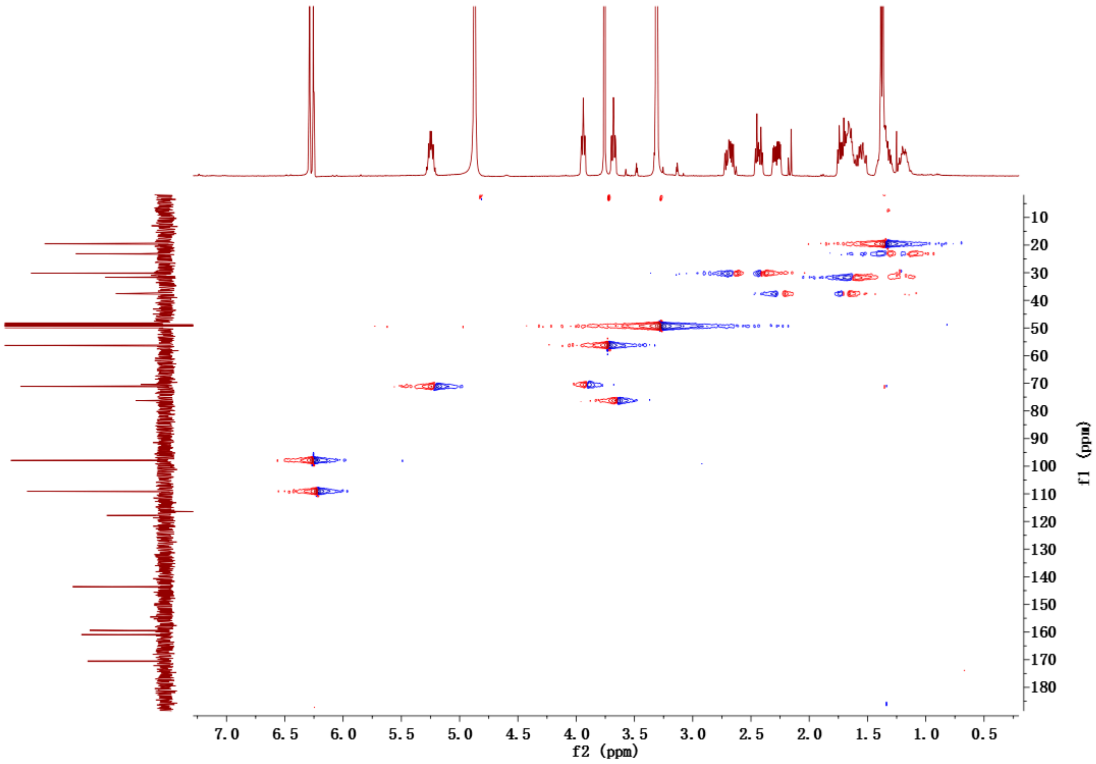

Figure S25. HMQC spectrum of compound **3** in CD3OD.

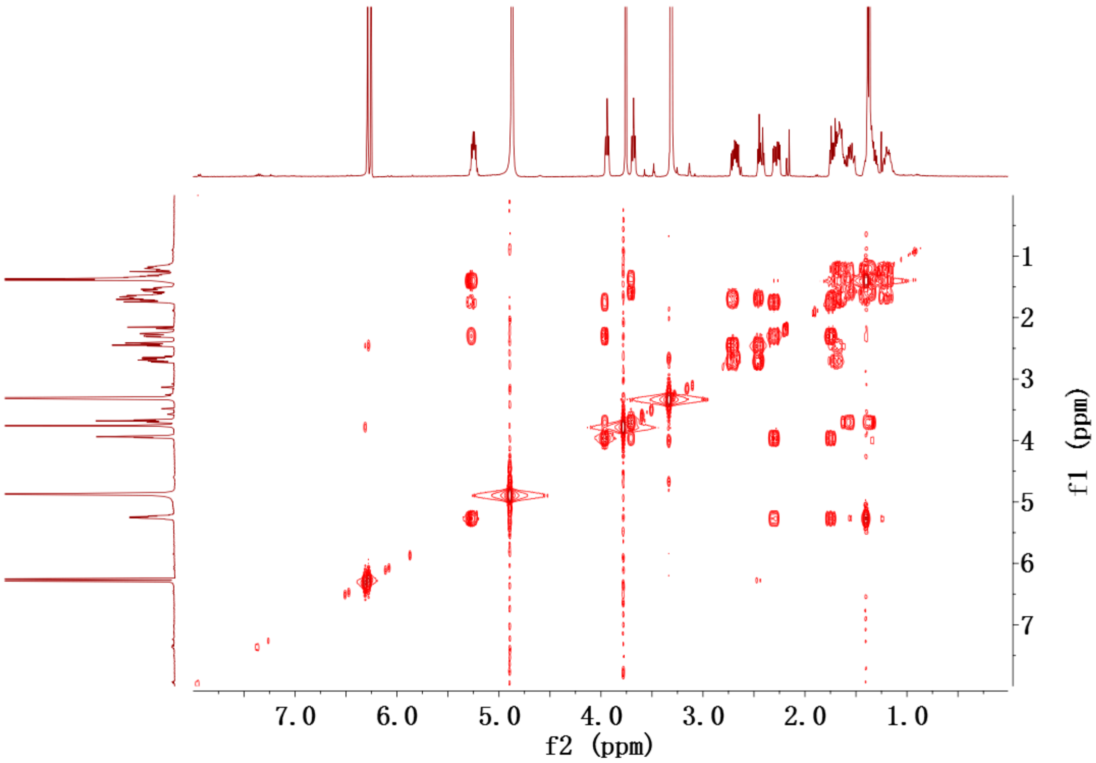

Figure S26. ^{1}H-^{1}H COSY spectrum of compound **3** in CD3OD.

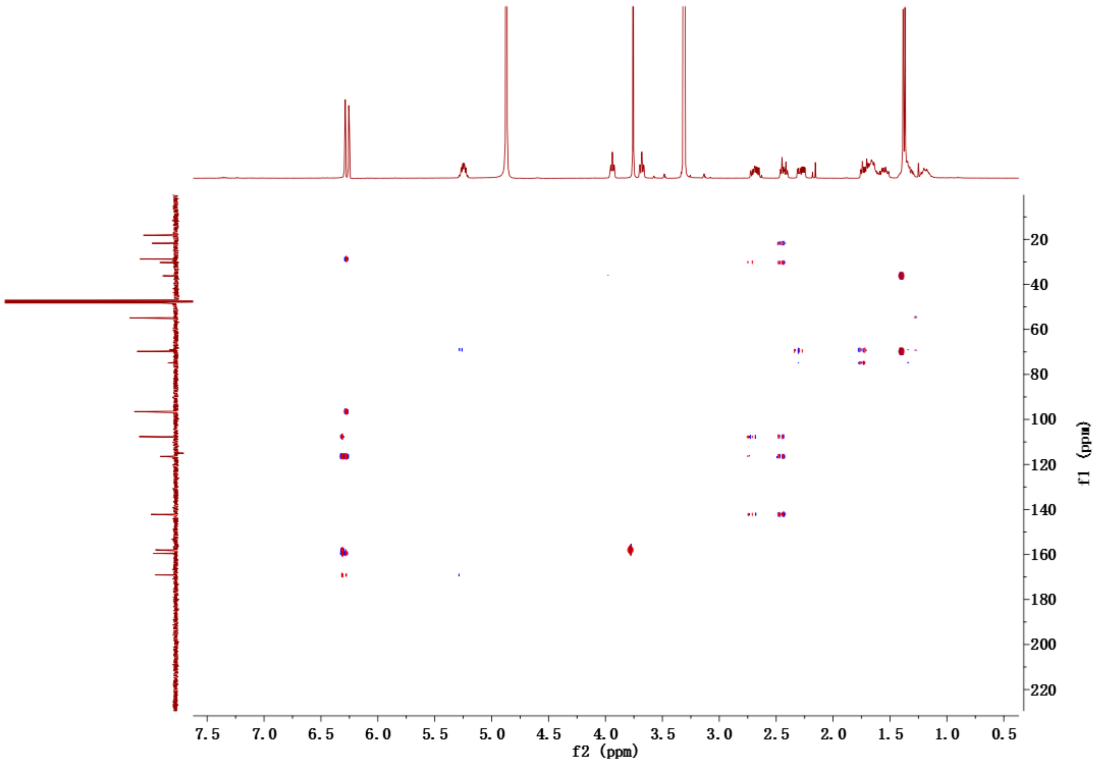

Figure S27. HMBC spectrum of compound **3** in CD_3OD.

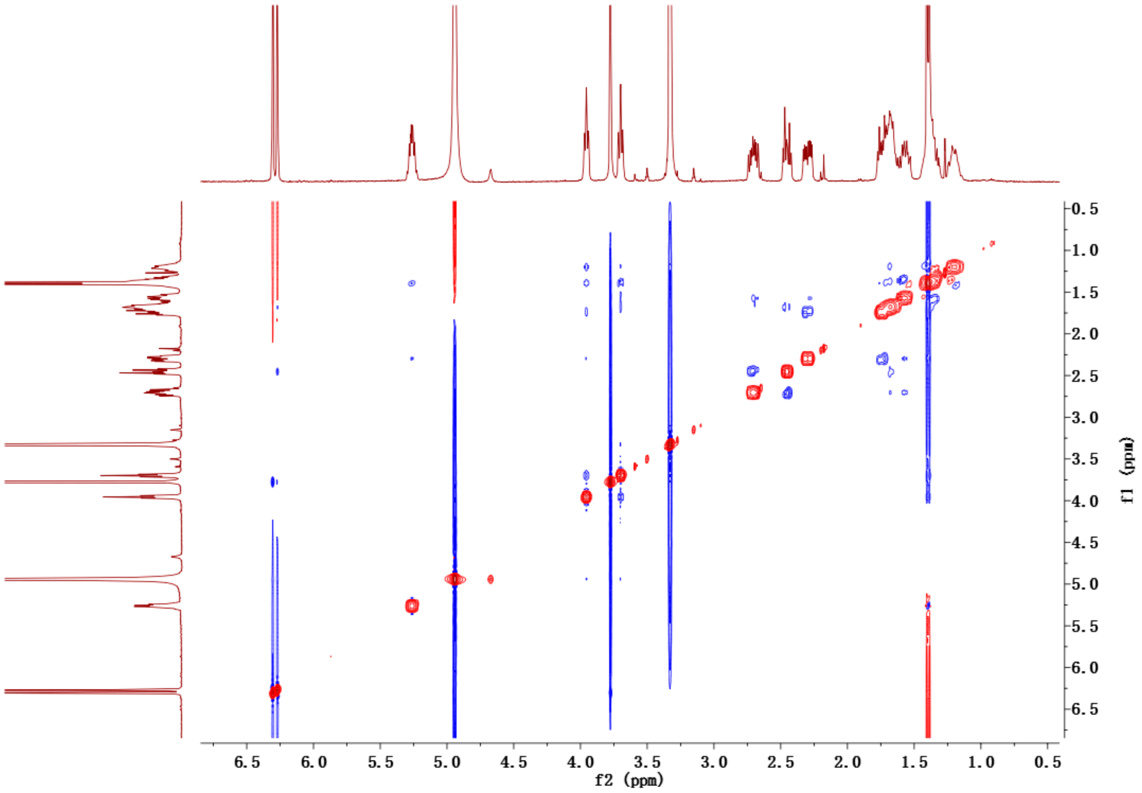

Figure S28. NOESY spectrum of compound **3** in CD_3OD.

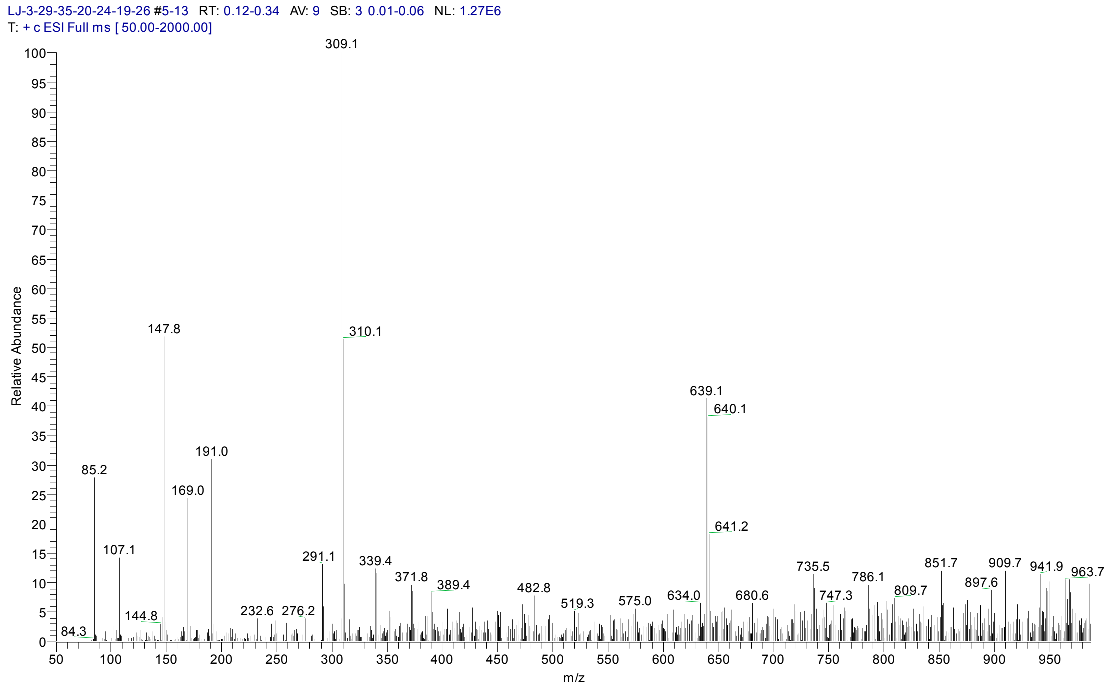

Figure S29. LR-ESI mass spectrum of compound **4**.

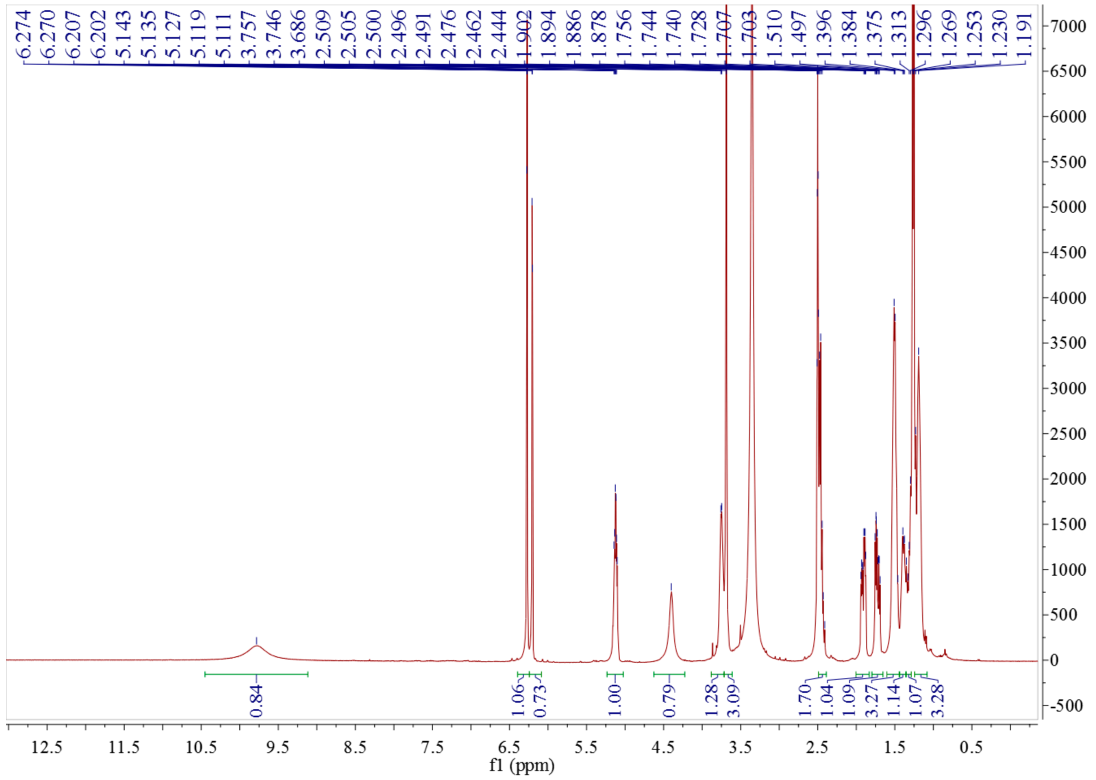

Figure S30. ^{1}H-NMR spectrum of compound **4** in DMSO-d_6 (400 MHz).

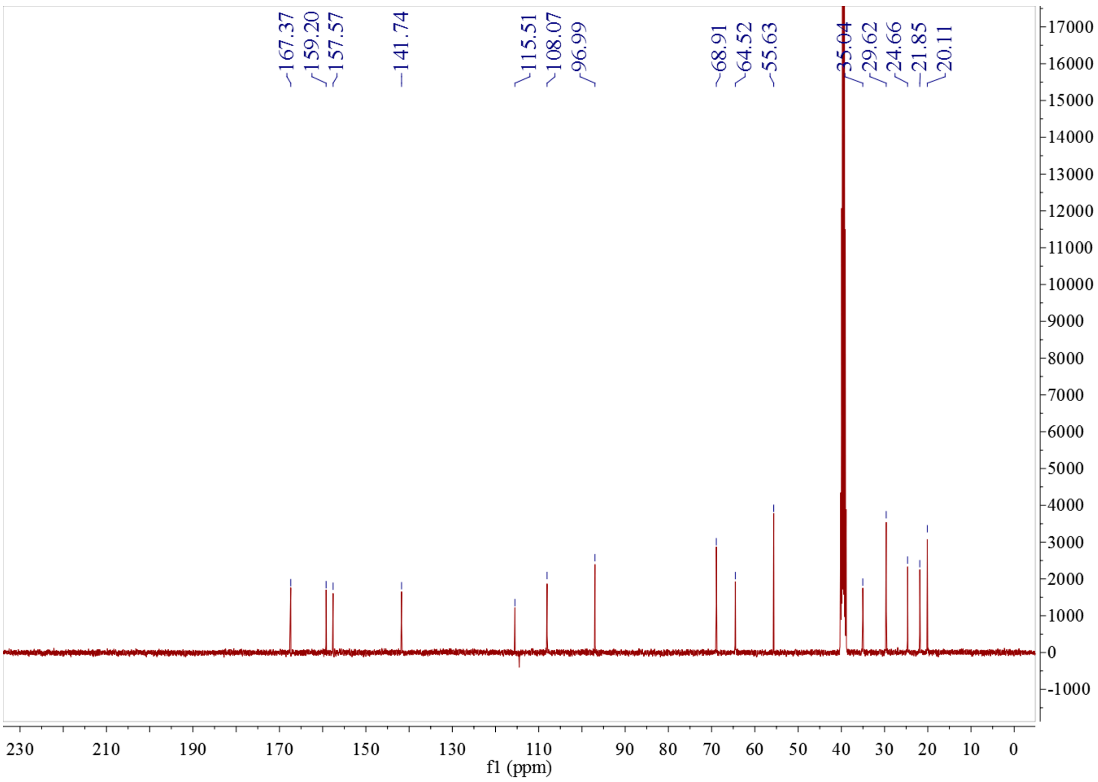

Figure S31. ^{13}C-NMR spectrum of compound **4** in DMSO-d_6 (100 MHz).

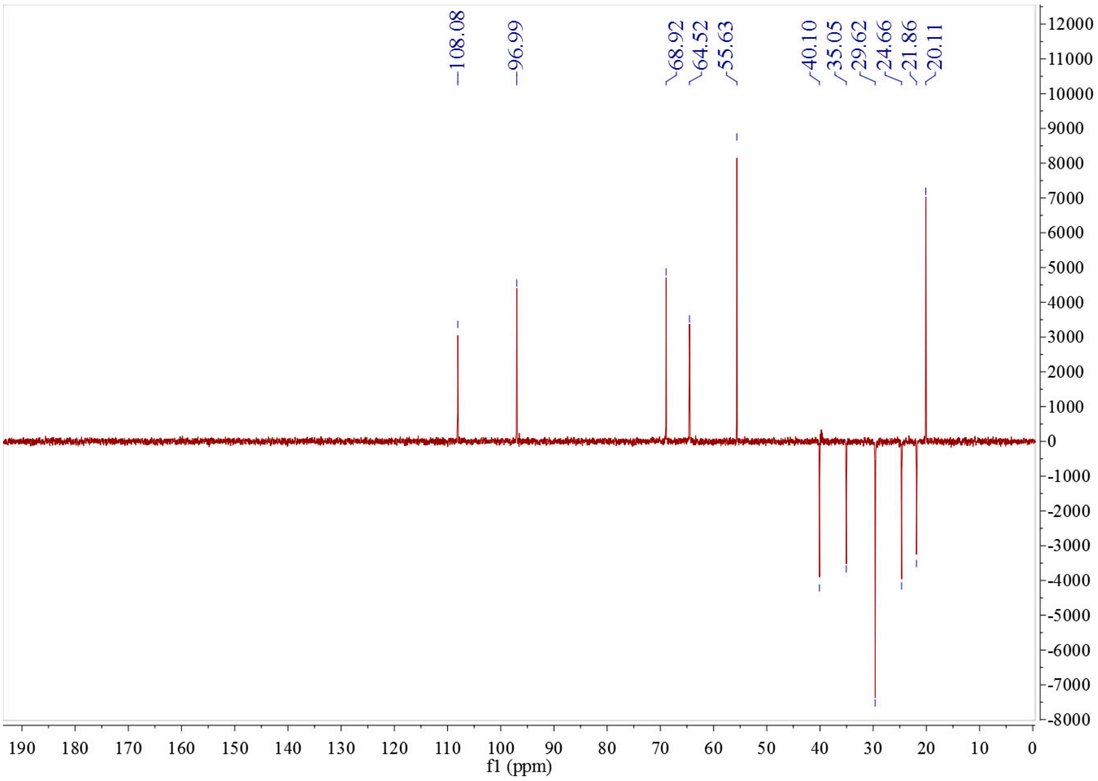

Figure S32. DEPT135 spectrum of compound **4** in DMSO-d_6.

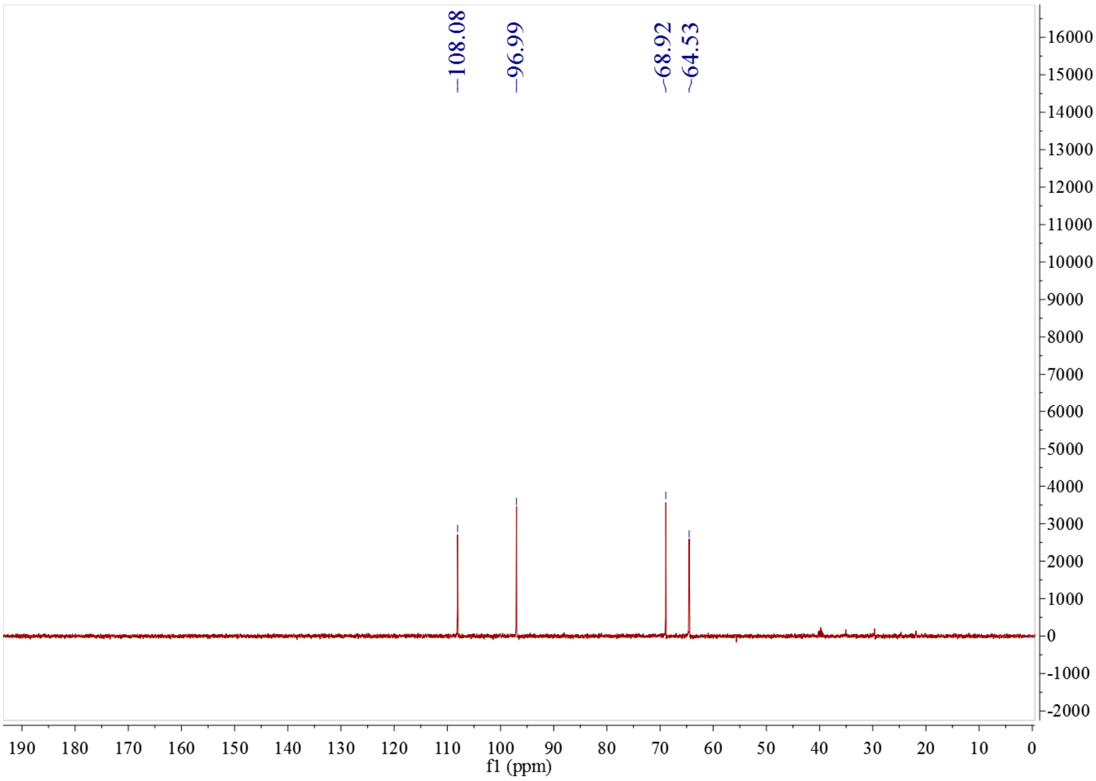

Figure S33. DEPT90 spectrum of compound **4** in DMSO-*d*6.

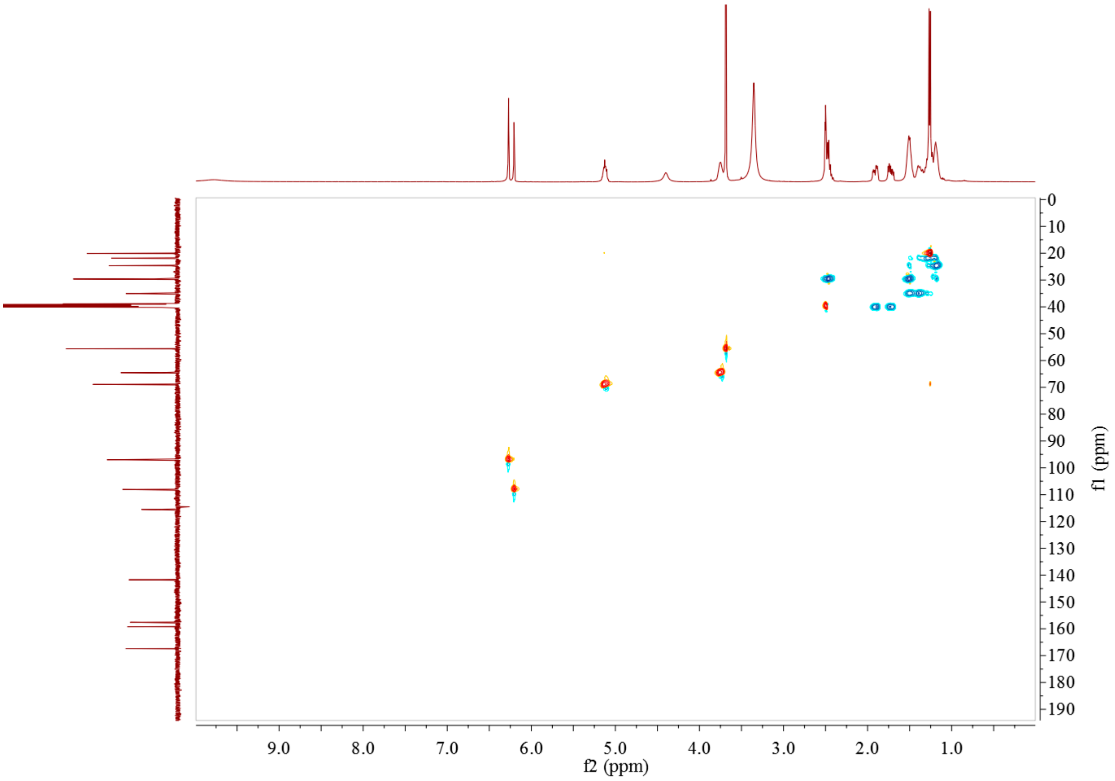

Figure S34. HMQC spectrum of compound **4** in DMSO-*d*6.

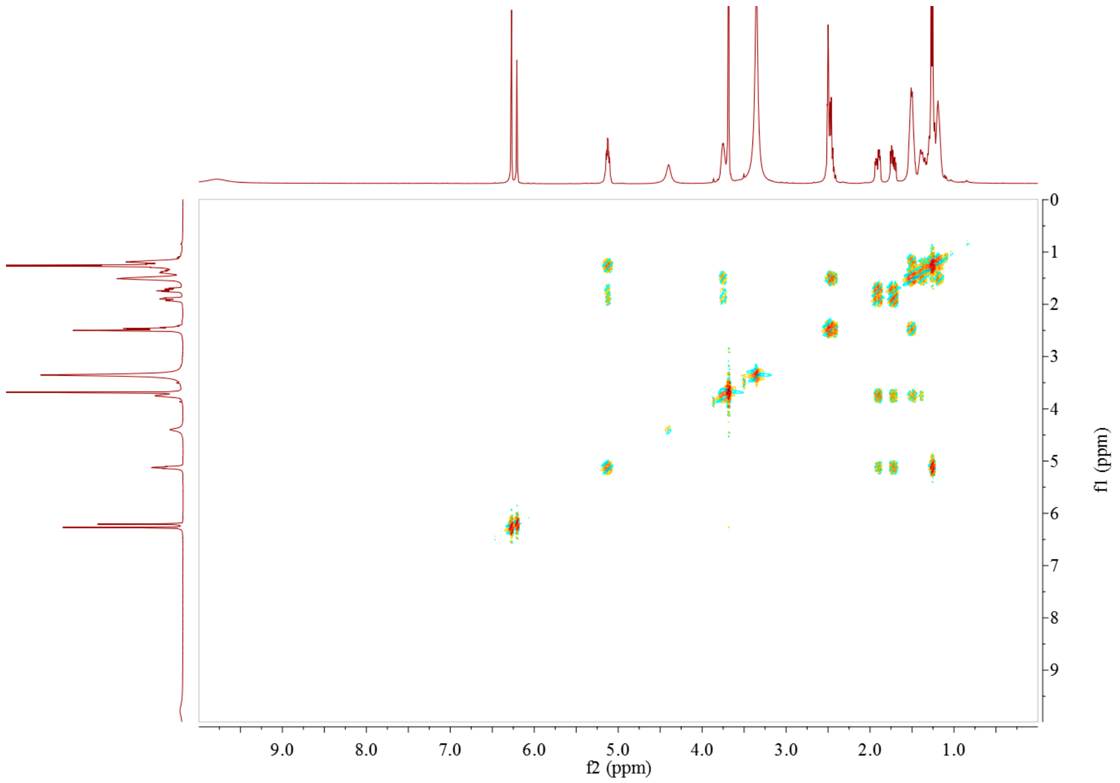

Figure S35. ^{1}H-^{1}H COSY spectrum of compound 4 in DMSO-d_6.

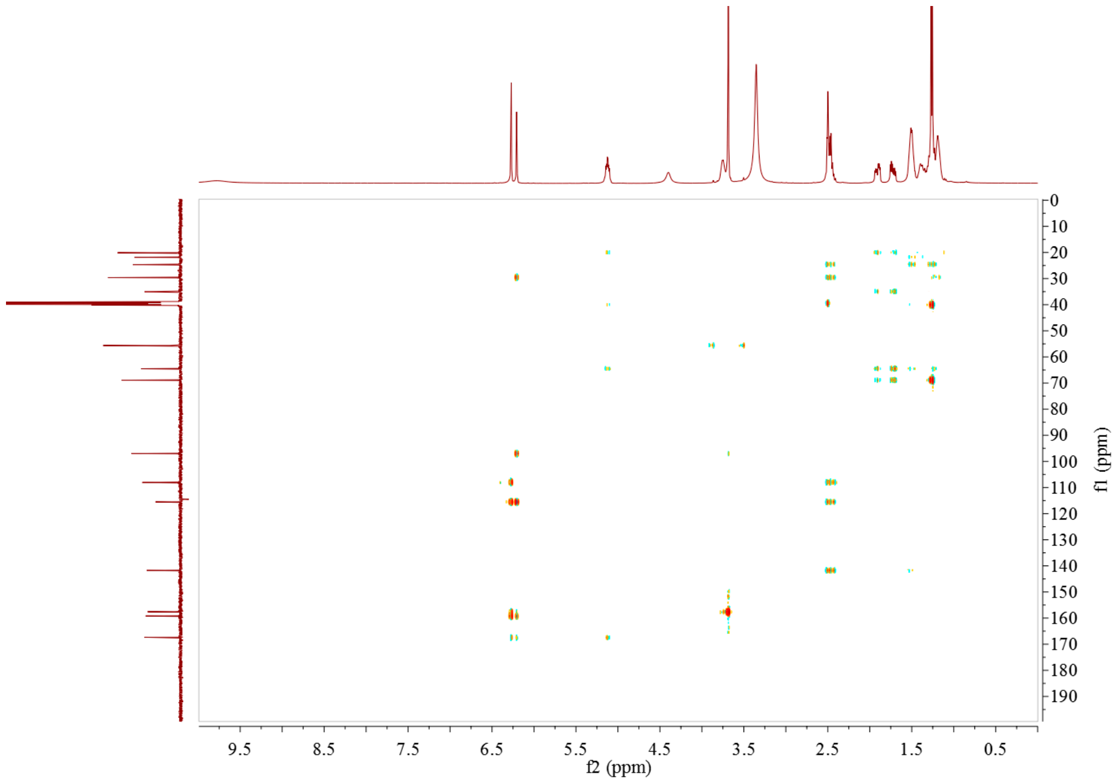

Figure S36. HMBC spectrum of compound **4** in DMSO-d_6.

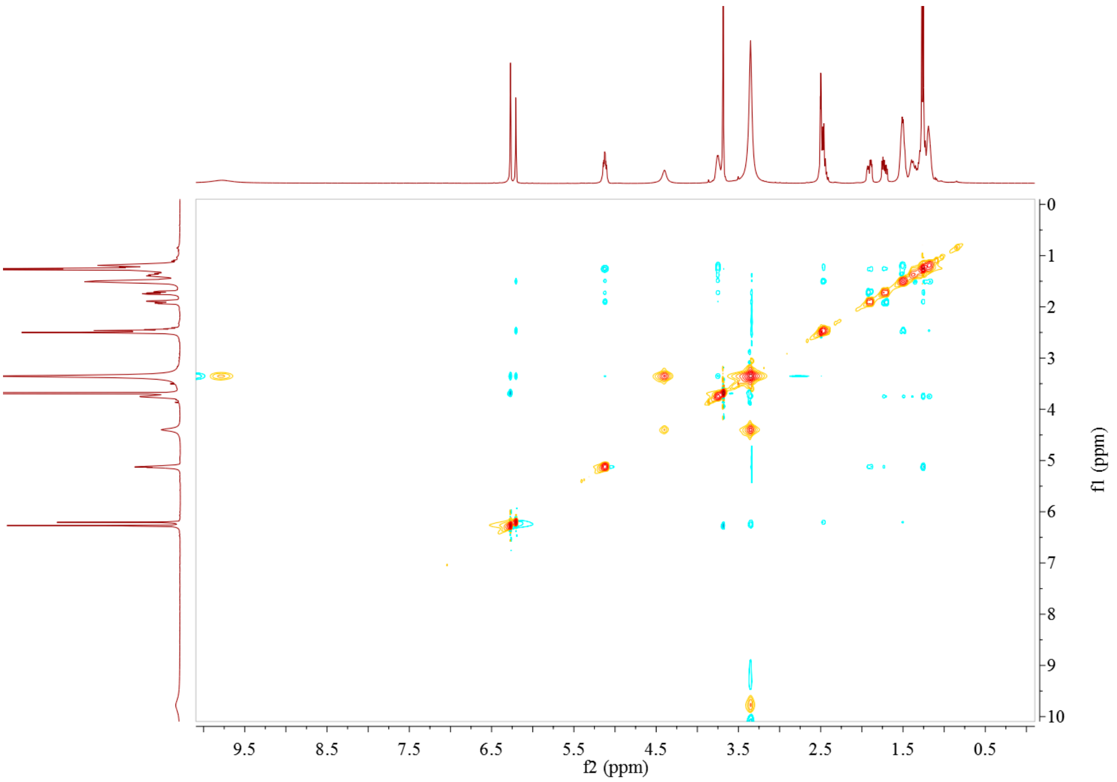

Figure S37. NOESY spectrum of compound **4** in DMSO-*d*6.

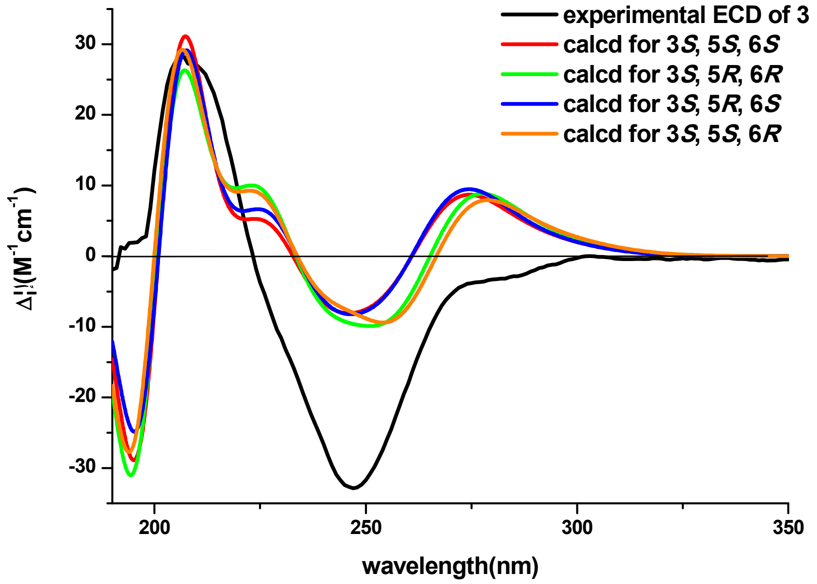

Figure S38. Comparison of the experimental ECD spectra of **3** with the calculated ECD spectra for four (3*S*) stereochemical options.

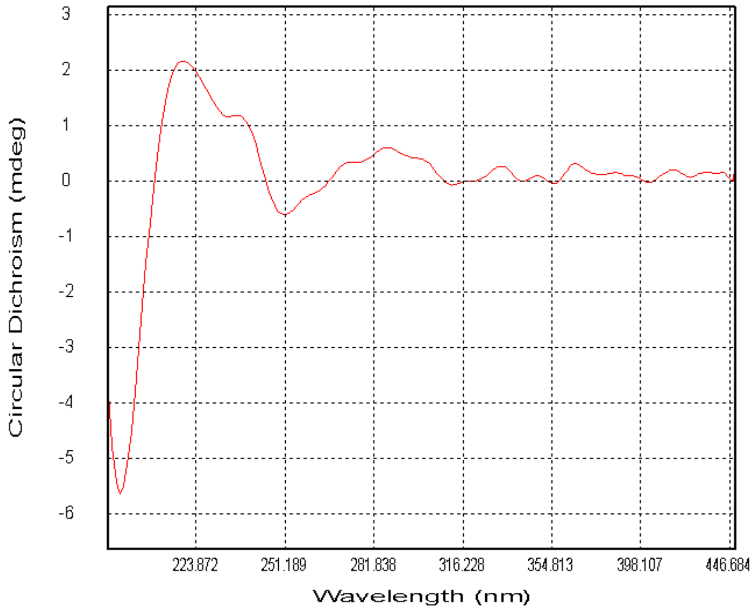

Figure S39. Experimental CD spectra of **2**.
